# Is Dysregulation of the HPA-Axis a Core Pathophysiology Mediating Co-Morbid Depression in Neurodegenerative Diseases?

**DOI:** 10.3389/fpsyt.2015.00032

**Published:** 2015-03-09

**Authors:** Xin Du, Terence Y. Pang

**Affiliations:** ^1^Mental Health Division, Florey Institute of Neuroscience and Mental Health, University of Melbourne, Melbourne, VIC, Australia; ^2^Behavioural Neurosciences Division, Florey Institute of Neuroscience and Mental Health, University of Melbourne, Melbourne, VIC, Australia

**Keywords:** HPA-axis, depression, cortisol, dexamethasone, Alzheimer’s disease, Parkinsonian disorders, Huntington’s disease, BDNFVal66Met

## Abstract

There is increasing evidence of prodromal manifestation of neuropsychiatric symptoms in a variety of neurodegenerative diseases such as Parkinson’s disease (PD) and Huntington’s disease (HD). These affective symptoms may be observed many years before the core diagnostic symptoms of the neurological condition. It is becoming more apparent that depression is a significant modifying factor of the trajectory of disease progression and even treatment outcomes. It is therefore crucial that we understand the potential pathophysiologies related to the primary condition, which could contribute to the development of depression. The hypothalamic–pituitary–adrenal (HPA)-axis is a key neuroendocrine signaling system involved in physiological homeostasis and stress response. Disturbances of this system lead to severe hormonal imbalances, and the majority of such patients also present with behavioral deficits and/or mood disorders. Dysregulation of the HPA-axis is also strongly implicated in the pathology of major depressive disorder. Consistent with this, antidepressant drugs, such as the selective serotonin reuptake inhibitors have been shown to alter HPA-axis activity. In this review, we will summarize the current state of knowledge regarding HPA-axis pathology in Alzheimer’s, PD and HD, differentiating between prodromal and later stages of disease progression when evidence is available. Both clinical and preclinical evidence will be examined, but we highlight animal model studies as being particularly useful for uncovering novel mechanisms of pathology related to co-morbid mood disorders. Finally, we purpose utilizing the preclinical evidence to better inform prospective, intervention studies.

## Overview

### Introduction

Mental illness has emerged to become one of the most significant chronic health issues at the present time. The management of the wide spectrum of psychiatric conditions presents as a great socioeconomic challenge. It is a multi-tiered problem ranging from early diagnosis and effective treatment of patients, counseling, and support for families to government-borne infrastructure and healthcare costs. Major depressive disorders (MDDs) are the third highest contributor to the global burden of disease (1, 2) and MDD is recognizably the most publicly discussed mental health condition. The combination of population growth and ever-increasing numbers of an aged population means that the absolute number of individuals suffering from depression is on a sharp upward trend (2, 3). A recent study conducted by the Centres for Disease Control and Prevention in the USA reported that of 235,067 adults examined, 9% were found to have symptoms of depression with 3.4% suffering from MDD. In Australia alone, an estimated one million adults suffer depression, with approximately one in six people expected to experience depression at least once in their lifetime (*Beyondblue*). Despite this high reported prevalence, the actual number of depression sufferers most likely exceeds estimates and predictions (4). Current research efforts have focused on increasing awareness for depression, better diagnostic approaches, and more effective treatment strategies. It is a leading cause of premature death in the US along with the likes of cardiovascular disease and cancer (5). It is estimated that over the next 20 years, depression will become the second leading cause of disability worldwide and the number one cause of disability in high-income nations, even discounting secondary diseases associated with depression (2, 3, 6–9). Depression is a major predictive factor for suicide (9) as it correlates with greater number of suicide attempts and increased lethality (10, 11). Research finds that 48% of depression patients have suicide ideation (12), and there is a significant correlation between number of depressive episodes and suicide attempts (13). It has been reported that compared with suicide attempters, suicide completers were more likely to be suffering from MDD (14, 15).

Depressed mood, in its own right, is a specific symptom of MDD (in accordance with DSM-5). The characteristic state of lowered mood and an aversion for participating in everyday activities collectively affects a person’s thoughts, feelings and wellbeing. One prevailing philosophy argues that depression is a result of the inability of the brain to make suitable adaptations in response to stressors due to impaired or inadequate neural plasticity (16–18). However, the pathophysiological factors underlying depression are numerous. The collective evidence suggests that all accounts of depression are unlikely to be associated with a single causative factor. Instead, it appears that depression manifests as a results of complex interplay between genetic (e.g., susceptibility genes) and non-genetic risk factors (e.g., traumatic life events).

One important consideration for medical practitioners and primary care providers is that while major depression comprises a major health concern in its own right, it is certainly not beyond appearing as a symptom of other health conditions. Depression is often reported as a co-morbid symptom in other neurological disorders, such as schizophrenia (19), Alzheimer’s diseases (AD) (20), Parkinson’s diseases (PD) (21, 22), and Huntington’s diseases (HD) (23–25). It is also present with major diseases, such as type II diabetes (26, 27), cardiovascular disease (6, 28), and alcohol withdrawal syndrome (29). In the context of cardiovascular disease, the presence of depression is a significant risk factor for worse outcomes and symptom severity [reviewed in Ref. (30–32)]. Thus, early recognition that the patient is exhibiting symptoms of depression should be followed-up with appropriate treatment so as to achieve a more wholesome recovery for the patient. To-date, there have been several publications discussing the implications of co-morbid depression relevant to these latter-mentioned health conditions, so this present review will focus on the neurological conditions.

Public education and advocacy for a better understanding of depression have had some success in pulling aside the veil of misunderstanding and prejudice associated with this mental health condition. While the social perception of this debilitating disorder has improved for the better, depression as a co-morbid symptom of neurodegenerative diseases, in particular AD, PD, and HD, has suffered neglect from the scientific and medical societies. Neurodegeneration is the umbrella term for loss of neuronal structure or functions. Depression is prevalent in all three diseases but has long been regarded by many as a collateral symptom caused by natural reaction to the decline of faculties, knowledge of diagnosis, or side effects of medications. Otherwise, it has been overshadowed by other symptoms, such motor and cognitive abnormalities. However, recent studies have provided evidence that depression within neurodegenerative disorders is possibly linked to the pathologies characteristic of these diseases. Better understanding of the etiology of depression in the context of the specific neurodegenerative disease will help us improve on current approaches to clinical treatment. Ultimately, it would lead to more effective and targeted treatments, which will undoubtedly benefit the patients and their families.

### The role of the HPA-axis in depression

The hypothalamic–pituitary–adrenal (HPA)-axis is the major endocrine system that regulates the physiological response to stress and as a result drive how an organism might adapt its own behavior or environment in order to accommodate that stress. The HPA-axis is well established to exert an influence over a wide variety of physiological processes including digestion, immune response, emotions, energy metabolism, and sexual behavior. The high degree of conservation in HPA-axis genes across species, even in the earliest vertebrates, is testament to its evolutionary significance (33). To briefly outline the HPA-axis circuit, the perception of a stress (e.g., threat to the life of the organism) initiates a signal within the paraventricular nucleus (PVN) of the hypothalamus. Neurons in the PVN synthesize and secrete corticotrophin-releasing hormone (CRH), which is shunted through the hypophyseal portal system and binds to specific receptors in the anterior pituitary (adenohypophysis). This then stimulates the synthesis and release of adrenocorticotropic hormone (ACTH) from the anterior pituitary, which is released into the circulatory system. Ultimately, ACTH triggers the synthesis and secretion of glucocorticoids (GCs; cortisol in humans, corticosterone in rodents) from the adrenal cortex (34). Activity of the HPA-axis may be altered with natural aging (35), but salivary (36) and plasma (37) cortisol concentrations are greater in individuals suffering from depression. Treatment with selective serotonin reuptake inhibitors (SSRIs) significantly decreases the concentration of urinary free cortisol levels (37). Separate reports have also demonstrated that HPA-axis activity is altered in AD (38), PD (39), and HD (40). Understanding HPA-axis pathology specific to these neurodegenerative conditions might shed light on why there is a higher incidence of co-morbid depression.

The HPA-axis exists as a self-regulatory physiological system responsive to negative GC feedback. GCs signal through two receptors, namely, the GC receptor (GR) and mineralocorticoid receptor (MR). The MR is a promiscuous receptor that binds, in addition to GCs, mineralocorticoids, aldosterone, deoxycorticosterone, and progesterone. However, in comparison, the affinity of GCs to MR is 10 times that of GR (41–43). The continuous MR activation by baseline levels of circulating GC is required for survival of dentate granule neurons (44) as well as having a plethora of functions, such as the regulation of electrolytes, blood pressure, and sympathetic drive (45, 46). GR, on the other hand, has low affinity for GC and is only active when GC levels are high, as in following the experience of a major stressor. The GR is involved in mobilization of energy resources and facilitates the termination of stress response and GC production, as part of the negative feedback loop to regulate HPA-axis activity (47). GR is highly expressed in the hippocampus, hypothalamus (48), and the prefrontal cortex (49). It acts as a ligand-activated transcription factor upon activation to control metabolism for fight-or-flight responses as well as inhibiting further GC production by suppressing HPA-axis activation (50, 51). Alterations of GC signaling play a major causal role in the development of depression (52, 53). These could be caused by subtle changes to GR function as a result of functional polymorphisms (54). The expression patterns of GR and/or MR are altered across different brain regions in AD (55) and PD (56). Given the important roles of GR and MR in regulating HPA-axis activity, it is therefore reasonable to speculate that a pathological alteration of GR/MR expression in those neurodegenerative diseases could contribute to the greater incidence of depression in these specific patient populations through a common modality, namely dysregulation of the HPA-axis.

Seminally, Carroll (57) found dysfunction of the HPA-axis in depression patients (57), giving a biological target to researchers who have since discovered the umbrella-like effects of this overarching endocrine system in relation to depression and psychiatric disorders in general. Clinically, as observed in Cushing’s syndrome, hyperactivity of the HPA-axis is highly correlated with significant increases in psychopathology, especially depression (58–60). Dorn et al. found in their study that 66% of Cushing’s patients exhibited psychopathology, consisting mainly of atypical and major depression as well as anxiety disorder and suicide ideation. Three months following treatment for hypercortisolemia, this dropped significantly to 54% and further decreased to 24% 12 months after correction (61). These suggested a direct causative link between elevated cortisol and psychopathology. Treatment of hypercortisolemia also reverses the hippocampal atrophy that is evident in Cushing’s disease (62). In clinical depression, hyperactivity of the HPA-axis is the most replicated biological state found (63, 64).

The dexamethasone suppression test (DST) is a method to evaluate HPA-axis function. It is based on the administration of dexamethasone (DEX), a synthetic GC that binds with high affinity to GR. This simulates the molecular cascade for negative feedback with the end result being a suppression of cortisol release. Blood is collected from the subject to determine their cortisol suppression response with the expectation that most individuals would have diminished serum/plasma cortisol levels. The DST has been used extensively in depression research, and non-suppression is observed with high frequency for individuals with major depression (65, 66) as well as other related conditions, such as bipolar disorder (67). It is important to note that DST non-suppression is also observed in a portion of the non-depressed population. The DST can be adapted to allow a more specific examination of the other key points of the HPA-axis. Several hours after the initial dose of DEX, CRH, or adrenocorticotrophin hormone (ACTH) is administered to the subjects prior to blood collection. The DEX–CRH combination allows evaluation of pituitary function with the cortisol response as the primary read-out, although ACTH levels can also be quantified (68, 69). In recovered patients, their DEX–CRH response does not differ compared to healthy controls (70). However, despite numerous patient–control studies and the suggestion that DEX–CRH is a more accurate approach to study HPA-axis activity (71), there is still an argument that the DEX–CRH test has yet to be fully validated as a clinical test for depression (72). In comparison, the DEX–ACTH combination is a directed examination of adrenal cortex function because ACTH interacts specifically with the adrenal cortical cells that respond by synthesizing and releasing cortisol. However, the take-up of this test has been very limited (73). Similarly, focusing on the neurological diseases, there are very few studies using the DST or DEX–CRH/ACTH tests (Tables 1–3). This is probably due to the fact that these tests are often used to probe the physiological aspects of depression symptoms, the latter of which tends not to be a priority for the treating physician. However, it is hoped that this will change given the mounting evidence that depression might be a significant modifier of disease process.

**Table 1:** **Summary of clinical and preclinical evidence for HPA-axis pathology in Alzheimer’s disease**.

Condition	Measurable		Directionality of change	Clinical/preclinical	Reference
AD	Cortisol levels	Basal serum/plasma	Increased	Clinical	(123, 126, 137, 143, 502, 503)
				Preclinical	TgCRND8 APP: (182); Aged male 3xTg-AD: (504)
			Unchanged	Clinical	(125, 134, 505, 506)
				Preclinical	Young 3xTg-AD transgenic line (male and female): (133, 507)
		CSF	Increased	Clinical	(55, 146)
				Preclinical	No preclinical evidence to-date
	Dexamethasone suppression test		Suppression	Clinical	(122, 508, 509)
				Preclinical	*apoE-/-* and ApoE ε3 transgenic lines: (510)
			Nonsuppression	Clinical	(124, 125, 506, 511, 512)
				Preclinical	ApoE ε4 transgenic line: (510)
	CRH challenge			Clinical	Hypersensitive cortisol response: (125, 502, 512); DEX-CRH challenge: hypo-response: (512)
				Preclinical	No preclinical evidence to-date
	ACTH challenge			Clinical	Hypersensitive cortisol response: (135); no change: (136)
				Preclinical	No preclinical evidence to-date

*Note the contradictory clinical evidence for the dexamethasone suppression test and limited evidence from animal model studies. Absence of preclinical evidence is noted for several measures*.

**Table 2:** **Summary of clinical and preclinical evidence for HPA-axis pathology in Parkinson’s disease**.

Condition	Measurable		Directionality of change	Clinical/preclinical	Reference
PD	Cortisol levels	Basal serum/plasma	Increased	Clinical	(280, 281, 286)
				Preclinical	TgS/MPTP pharmacological model: (513)
			Unchanged	Clinical	(287, 291)
				Preclinical	8-OH-DPAT/l-Dopa pharmacological model: (514)
			Decreased	Clinical	(276, 279)
				Preclinical	No preclinical evidence to-date
		CSF	No data available		
	Dexamethasone suppression test		Suppression	Clinical	(39, 508)
			Non-suppression	Clinical	(286)
	CRH challenge			Clinical	No clinical evidence to-date
				Preclinical	No preclinical evidence to-date
	ACTH challenge			Clinical	Normal (cross-sectional study with no healthy controls): (515)
				Preclinical	No preclinical evidence to-date

*Note the unavailability of clinical data regarding CSF cortisol levels and responses to CRH or ATCH challenges. Also note the relative lack of depth to the preclinical evidence here compared to AD and HD*.

**Table 3:** **Summary of cortisol changes detected in Huntington’s disease and other functional tests of HPA-axis regulation**.

Condition	Measurable		Directionality of change	Clinical/preclinical	Reference
HD	Cortisol levels	Basal serum/plasma	Increased	Clinical	(40, 399, 400, 407)
				Preclinical	6-week R6/2 transgenic line: (402)
			Unchanged	Clinical	Single cohort of female patients: (383)
				Preclinical	12-week R6/1 transgenic line: (412)
			Decreased	Clinical	Specific to non-depressed patients: (403)
		CSF	No data available		
	Dexamethasone suppression test		Suppression	Clinical	(399, 410)
				Preclinical	(412)
			Non-suppression	–	
	CRH challenge			Clinical	Greater cortisol peak: (399)
				Preclinical	Normative corticosterone peak in DEX-CRH challenge: (412)
	ACTH challenge			Clinical	No clinical data available
				Preclinical	Elevated corticosterone peak in DEX-ACTH challenge: (412)

*Note the absence of preclinical evidence from other rodent models of HD besides R6 transgenic mouse lines*.

### Other molecular pathologies linked with HPA-axis dysfunction

Tight regulation of GC levels is essential as prolonged exposure to high concentrations alters numerous cellular processes potentially damaging the brain. Neuronal activity is directly influenced by GC levels through its regulation of the alpha subunit of the active Na+ channel (74). Cell turnover in the hippocampus is generally inhibited by higher levels of GCs, and numerous studies have found decreased numbers of proliferating cells and reduced cell survival in the dentate gyrus after administration of corticosterone in rodents (75–77). Chronic exposure to high levels of GCs is also linked with apoptosis, particularly of the GR-expressing neurons in the hippocampus (78–81). Administration of high doses of GCs led to atrophy in the hippocampus of rats (82) and monkeys (83), as well as neuronal atrophy (84) and volume reduction in the prefrontal cortex (85). These effects are attributable to the regulatory role of GR in increasing the ratio of pro- versus anti-apoptotic molecules as demonstrated in the rat hippocampus (86). GR activation also induces translocation of the tumor suppressor protein to the nucleus thereby increasing its transcriptional activity (87).

Depression is notoriously plethoric in its various aspects of pathology. Numerous molecular signaling systems, such as monoamines like serotonin, noradrenalin, and dopamine are found to be dysregulated (88–90). Inflammatory cytokines are abnormally increased in depressive disorder (91). Brain-derived neurotrophic factor (BDNF), an essential neurotrophin, is reportedly diminished in depressed patients but recovered in patients receiving treatment (92). Separately, altered BDNF levels and signaling through the TrkB receptor have been implicated in the pathophysiology of PTSD (93). Interlaced with all the aforementioned factors is the HPA-axis which influences and is in turn influenced by monoamines (94–97), cytokines (98–100), and BDNF/TrkB signaling (101).

### Rationale for this review

Separately, there are good bodies of evidence supporting a higher incidence of depression and HPA-axis dysfunction in AD, PD, and HD. However, whether the disease-related HPA-axis dysfunction accounts in some part for the co-morbid depression remains unclear. It will be important to consolidate our knowledge of how disease-specific events bring about HPA-axis dysfunction through molecular pathology, and how the consequentially altered levels of circulating GCs (especially in the brain) might impact on disease progression and severity of symptoms. In this review, we will summarize the current literature regarding the prevalence of co-morbid depression in AD, PD, and HD. We will then examine the evidence of HPA-axis dysfunction in the three neurodegenerative diseases, drawing not only on the findings of clinical studies but also preclinical evidence where available. The DST will be discussed as a potentially reliable clinical tool to identify patients manifesting HPA-axis dysfunction who might then be at risk for developing depression. We will also consider more recent evidence of how GCs might change the processing or interactions of key disease-related proteins (e.g., Aβ, htt) and how this might affect disease progression.

A discussion of the similarities and differences underlying the system and its regulation and influences upon factors intimately linked with depression in the various milieus of AD, PD, and HD may prompt new avenues through which to tackle depression in neurodegenerative diseases.

## Depression in Alzheimer’s Disease

Alzheimer’s disease is the leading cause of dementia and its etiology comprises a complex interaction between multiple genetic risk factors and environmental (lifestyle) modifiers (102, 103). There are two broad classifications of AD – early and late-onset. Late-onset AD is generally linked with aging; although recent studies have uncovered the involvement of significant genetic influences too (104). In addition to specific impairments of memory recall as part of a broad dementia spectrum, psychiatric symptoms are also a prominent feature of this disease.

Symptoms of MDD are reported more frequently in AD patients compared to the wider healthy population. The presence of depression significantly influences AD brain pathology (105), including increased accumulation of amyloid protein in the brain (106). Vascular risk factors and altered neurotransmitter signaling have also been put forth as causative factors for co-morbid depression [reviewed by Chi et al. (107)]. However, the precise pathophysiology that accounts for the behavioral symptoms has not been determined. Interestingly, both independent studies and meta-analyses have reported that depression is a significant risk factor for developing AD (and other forms of dementia) (108, 109). Consistent with that, recent re-examination of the data from the Framingham Heart study found that depression was a significant risk factor for dementia and AD in older males and females within that study population (110). For some, these psychiatric changes may develop prior to cognitive decline. In a recent review, Belleville et al. suggested that the presence of neuropsychiatric symptoms including depression and anxiety should be included as significant factors in a multi-factorial predictive model for the earliest signs of AD (111). There is evidence that depression co-morbidity is associated with a greater extent and progression of disease pathology, such as increased neurofibrillary tangle load (112) and poorer rate of cognitive decline (113). It is quite concerning that a recent meta-analysis revealed a lack of efficacy of typical antidepressant medications for treating AD patients for co-morbid depression (114). The molecular and cellular effects of antidepressant compounds are well understood, so further research should be invested into understanding why there is this apparent lack of efficacy of these drugs to treat the depressive symptoms. The current evidence also indicates that effective treatment of co-morbid depression with SSRIs is not associated with an improvement of cognitive symptoms (115). That implies that the cognitive aspects of AD symptomatology are separate from depression pathology, but it is still possible that the benefits of antidepressant treatment may manifest in other markers of pathology. It is worth noting that MDD is defined by a range of symptoms, and further research is still required to parse out the specific aspects of MDD that may relate more specifically to AD pathology (116). To that effect, one recent study has suggested the specific behavioral symptoms may differ for AD and mild cognitive impairment (117).

Studies of preclinical models of AD have comprehensively demonstrated that depressive symptoms are almost certainly part of the wider pathological phenotype of the disease. Studies of rodent behavior are unlikely to recapitulate the psychosocial aspects of AD, so any changes in the behavioral phenotype are more than likely attributable to *in vivo* pathophysiology. A single intracerebroventricular (i.c.v.) injection of soluble oligomers of the amyloid-β peptide elicits pro-depressive behavioral changes in mice after 24 h (118). That effect on behavior was not only acute, but also persisted for 8 days (perhaps linked to protein turnover). Treated mice displayed greater immobility time on the forced-swim test, a well-validated behavioral test used to quantify behavioral despair. Mice also exhibited reduced exploratory movement (hypolocomotion) and lower preference for sucrose-sweetened solution (anhedonia – another key symptom of depressive disorder). Treatment with the SSRI fluoxetine was capable of rescuing the forced-swim test phenotype, although its effect in the other tests was not established in this study. Previously, another study had administered the Aβ(1–40) peptide i.c.v. and also reported that treated mice recorded increased FST immobility times that were rescued by acute desipramine injection (119). A separate study using a similar approach also reported increased immobility time on the tail-suspension test, and that behavioral response was blocked by pre-treatment of the neuropeptide NPY (120). In future, it would be interesting to investigate using preclinical models whether drugs commonly prescribed to AD patients to treat the cognitive symptoms of dementia (e.g., acetylcholinesterase inhibitors or memantine) are also effective in modifying depression-related behaviors.

### HPA-axis pathophysiology mediates depression in AD

The collective evidence strongly indicates that activity of the HPA-axis is dysregulated in AD. The presence of the pathological soluble form of Aβ alone appears to be the key event sufficient to deregulate central control of HPA-axis activity. In rats, the single action of an acute i.c.v. injection of Aβ(25–35) is sufficient to induce HPA-axis hyperactivity (121). Therefore, in humans, the early accumulation of the pathological forms of Aβ is likely to contribute to overall dysregulation of the HPA-axis. This is supported by evidence that early-stage AD patients have increased basal plasma cortisol levels (122, 123) and decreased sensitivity to low-dose DEX suppression (124, 125). In mild to moderate AD, serum cortisol levels remain significantly elevated together with DHEA and androstenedione levels, while estradiol levels were unaffected in females (126). The majority of investigations to-date have tended to focus on the central premise that there is hyperactivity of the HPA-axis in AD without more closely examining HPA-axis pathology beyond the hypothalamus. Few studies have explored beyond measuring cortisol as a direct reflection of HPA-axis activity. There are numerous targets one could quantify as a measure of HPA-axis activity including hypothalamic neuropeptides and androgens. This is one aspect of the AD research field that could be improved upon.

One of the earliest knowledge of HPA-axis dysfunction in AD was from the correlation of cerebrospinal fluid (CSF) CRF-like immunoreactivity with the patients’ neuropsychological ratings (127) and severity of dementia (128). However, no significant difference in serum CRF levels was reported for AD patients and health controls (129). Moreover, CRF changes are likely to be central, not peripheral, because subsequent closer inspections of post mortem AD brains revealed decrease in both free and complexed forms of CRF in a variety of discrete brain regions (130). These suggested that in AD, either hypothalamic CRF-secreting neurons are hypoactive or there is an active modulation to reduce CRF tone in response to HPA-axis hyperactivity. There is some evidence of AD pathology impacting on CRF-immunopositive neurons within the PVN of the hypothalamus (131, 132). The human post mortem data are complemented by findings of early diminishment of CRF gene expression in PVN neurons in presymptomatic 3xTg-AD mice (133). Interestingly, the downregulation of CRF gene expression could be a homeostatic response to HPA-axis hyperactivity because AD patients administered a dose of CRF have a significantly greater cortisol response compared to healthy controls (134). At a glance, the progressive pathology within the hypothalamus has not been thoroughly examined in the AD brain. Similarly, the evidence of AD-associated adrenal dysfunction is few and even contradicting. One study had reported adrenal hypersensitivity after finding significantly greater cortisol response to ACTH stimulation (135), but this was contradicted by a second study that instead claimed abnormal androgen secretion (136).

Alzheimer’s disease patients have significantly higher levels of cortisol with an exaggerated diurnal rhythm (134, 137). Consistent with that, cortisol levels quantified from ventricular CSF of pre-senile AD patients post mortem are significantly higher compared to age-matched controls (138). The directionality of that change is identical to what is observed in MDD (as mentioned in Introduction). Recent studies by Peavy et al. suggest that the initial signs of dementia-associated cognitive changes are reflective of HPA-axis dysregulation. Using a series of multivariate logistic regression analyses to qualify the self-reported complaints of memory deficits by cognitively normal elderly volunteers, Peavy et al. reported that both the average post-peak cortisol level and the cortisol awakening response correlate with the number of memory-related complaints (139). However, dysregulation of the HPA-axis itself does not appear to directly contribute to the worsening of disease symptoms because those same measures of cortisol were not associated with the progression from mild cognitive impairment to dementia (140). Instead, it is highly probable that other environmental modifiers, such as a persistent exposure to stress, influence the rate of cognitive decline (141). The impact of stress on cognitive function is well established. It is not surprising that HPA-axis dysfunction can cause cognitive deficits because the hippocampus, which is central to memory and cognition [reviewed by Opitz (142)], is also highly dense in GR expression and closely linked to depression pathology. Interestingly, a study by Murialdo et al. examining the relationship between hippocampal dysfunction and HPA-axis found that dehydroepiandrosterone (DHEAS) levels were a better correlate than cortisol levels for impaired hippocampal perfusion measured using SPECT imaging (143). Perhaps future studies of hippocampal dysfunction as a structural surrogate of cognitive deficits and depressive disorder should explore the inclusion of both cortisol and DHEAS levels as part of their predictive modeling.

Serum and plasma cortisol measurements have previously been suggested as a reliable biomarker for AD (144, 145). Additionally, the well-described e4 risk variant of apolipoprotein (APOe4) risk genotype further distinguishes a subpopulation with significantly higher plasma cortisol levels (55, 146, 147), a likely reflection of the role that the APO protein has in the normal regulation of GC synthesis (148). However, it has also been suggested that the APOe4 genotype is predictive of central nervous system (CSF) cortisol levels but not peripheral (serum) cortisol levels (149). There is a current lack of information regarding the accuracy of central (compared to peripheral) cortisol levels as a biomarker for AD, partly attributable to the preference and ease of collecting blood samples from patients compared to lumbar puncture. However, in spite of the procedural challenges, it is important that we establish which biosample more accurately reflects APOe4-associated cortisol levels, and resolution of this matter can only be achieved by future studies utilizing large patient cohorts. Knowing how the APO genotype influences HPA-axis dysfunction will also be important for understanding how this genotype modulates cognition. Although Peavy et al. reported that the well-described APOe4 could not account for the greater number of memory-related complaints in their study (139), a separate study has claimed that the APOe4 is associated with worse baseline memory performance and an accelerated rate of memory decline (150). It is possible that this inconsistency reflects the reliance on self-reported data as well as the use of different patient questionnaires, so the findings of these studies cannot be regarded as conclusive until successfully replicated by independent research groups.

Genetic heterogeneity presents as an enormous hurdle to population-based clinical studies seeking to uncover subtle phenotypic differences. Large cohort studies will be required to account for the likely involvement of multiple AD risk genes acting as “genetic triggers” for HPA-axis dysfunction and depression. In this regard, preclinical studies of rodent models that are genetically homogeneous have tended to be more useful for understanding the roles of specific AD risk genes in the disease phenotype. Young asymptomatic 3- to 4-month-old 3xTg-AD mice possessing the PS1M146V, APPswe, and tauP301L transgenes (151) have normal basal levels of corticosterone (133). While hippocampal dysfunction is already evident in these young animals (152), the depressive behaviors only emerge as the mice age to 20 months (153). A similar discordance has been reported in a separate transgenic mouse model which over-expresses human APP wherein impaired hippocampus neurogenesis was evident months prior to the emergence of depressive behavior (154). For both transgenic lines, whether the depressive phenotype emerges concurrent with HPA-axis dysfunction is unknown. Future studies should examine this so as to generate preclinical evidence that dysfunction of the HPA-axis is causative of the depressive symptoms. In addition, such a study would uncover any potential relationship of those AD risk genes with HPA-axis dysfunction and the depressive symptoms.

Studies of rodent models of AD have yet to thoroughly investigate the potential of environmental enrichment, an experimental paradigm well established to exert anxiolytic, anti-depressive, and pro-cognitive benefits [see review by Nithianantharajah and Hannan (155)]. A recent study demonstrated that environmental enrichment rescues the cognitive deficits in the Tg2576 transgenic mouse model of AD by reducing levels of tau phosphorylation and rescuing the deficit in hippocampal neurogenesis during disease progression (156). Similarly, despite strong evidence that physical activity is a significant modifier for dementia-related cognitive deficits [reviewed by Pang and Hannan (157)], few studies have examined the potential to rescue depressive behavioral changes in the context of AD (and more broadly, dementia). Thus, investigating the anti-depressive effects of environmental enrichment and physical activity on the various transgenic mouse models of AD would appear to be a worthwhile endeavor.

### Molecular pathologies of the HPA-axis in AD

Multiple genetic risk factors implicated in MDD could be modulating the risk for co-morbid depression within the AD population. The combination of two or more gene × gene interactions wherein each gene polymorphism subtly alters a given signaling pathway culminates in a significant neurochemical imbalance and the higher risk for developing depression. Genetic polymorphisms of a number of serotonin receptors well established to be involved with MDD pathology have been independently linked to the increased likelihood of AD patients developing symptoms of depressive disorder (158). A separate genetic risk factor, the BDNF Val66Met polymorphism, has been reported to stratify AD patients with and without co-morbid depression (159). The BDNF Vall66Met functional polymorphism was also implicated in a more recent cross-sectional study wherein sex was an additional factor of investigation (160). BDNF signals downstream via the TrkB receptor and selective receptor agonists such as 7,8-dihdyroxyflavone are well established to exert anti-depressive effects (161, 162). Several studies have established the neuroprotective effects of 7,8-dihdyroxyflavone on cognitive deficits in mouse models of AD (163–166). However, its effectiveness in rescuing the depressive phenotype has yet to be investigated. Arlt et al. reported that there was an increased risk of having MDD for female AD patients with the BDNF Val66Met polymorphism (160). This was one of a few studies that had identified gender as a significant modifier for disease symptoms. Further investigations should be conducted to examine potential sex-specific differences in neuronal pathology within the brain regions that are known to regulate emotionality. Three other significant genetic polymorphisms were also identified in that study of which only the FKBP5 polymorphism was significant for their entire cohort. FKBP5 has been strongly implicated in depression pathology (167–169), so future studies should investigate whether FKBP5 polymorphisms are also significant modifiers for depression within the AD patient population. For the purpose of this section, we will specifically focus on molecular pathology within the HPA-axis attributed to genetic risk factors for AD and how this might impact on depressive symptomatology.

Having the knowledge of the longitudinal progressive molecular pathology of an AD brain paired to the precise moment at which specific disease symptoms manifest would be ideal. Unfortunately, it is practically impossible to conduct clinical studies to identify the earliest molecular pathology in AD brains and track their natural progression. This can be achieved with preclinical models with the one caveat that current rodent models of AD are based on infrequent/rare genetic mutations identified in the human population. Thus, there is somewhat limited construct validity. Recently, using a transcriptomics approach, a profiling study of brains collected from APP/PS1 transgenic mice at different ages revealed that the earliest detectable disease-related changes (as opposed to age-related changes) are the downregulation of HPA-axis-associated genes linked to metabolism, depression, and appetite (170). This finding strongly supports the hypothesis that dysregulation of the HPA-axis is a key pathological event in early AD. In fact, it could be the seed event for other aspects of disease pathology. It also provides a pathophysiological basis for the higher incidence of co-morbid depression in AD, especially as one of the earliest predictive symptom of AD. However, a major short-coming of that study was that it did not examine depression-associated behavioral changes in the mice at those same ages. Progressive behavioral changes have previously been described in the APP/PS1 transgenic mouse model (171) but there has yet to be a study focusing on depression-related behaviors. A longitudinal study of that nature that tracks the development of behavior symptoms correlated to molecular pathology in specific brain regions would provide great insight into development of depression in AD. However, returning to the current limitations of preclinical models of AD, the heterogenetic nature of this disease means that those findings would be limited to APP/PS1 mutations, and subsequent validation studies using other genetic models would still be required.

While there is clinical evidence that the APO genotype might predict hippocampal-dependent cognitive deficits, a key question that remains unresolved is whether APOe4 similarly predicts the likelihood of an AD patient developing depressive symptoms. At present, this is unclear. Locke et al. claim that this risk genotype does not affect the incidence and the progression of depression symptoms (172) but this lone study still requires independent replication in larger cohorts. A close relationship between the APOe4 risk genotype and depression in the context of AD was uncovered by a recent functional MRI study (173). It was reported that the APOe4 risk genotype and presence of geriatric depression were significant co-variables in accounting for changes in cortico-hippocampal connectivity resulting in a greater risk for developing AD. That is, having depression worsens the extent of structural pathology in the AD brain. However, with recent reports of novel AD-associated gene loci (104, 174), the likelihood of a predictive model for depression in AD based on a single geneotype is slim. That notion is supported by the knowledge that the extent of APOe4-linked HPA-axis dysfunction is further subject to additional genetic modifiers, e.g., TOMM40, a variable-length polymorphism that exists in linkage disequilibrium with APOE (175, 176). In other words, co-morbid depression might manifest differently in specific subpopulations of AD patients, requiring them to carry a specific combination of risk genes. As evidence of this, de Quervain et al. (177) reported that a rare haplotype in the 5’ regulatory region of 11beta-hydroxysteroid dehydrogenase type 1 (HSD11B1) was associated with increased risk for sporadic AD (177). The functional consequences of altered HSD11B1 on overall HPA-axis activity in the presence of Aβ pathology have yet to be studied. Thus, future studies will have to discard a monogenic associative approach and expand to consider the influence of multiple genes in order to accurately determine the incidence of co-morbid depression in AD.

The GR is the essential regulator of the HPA-axis negative feedback system, and several SNPs have been linked to MDD pathophysiology, metabolic changes, and dysregulation of stress response [reviewed by Manenschijn et al. (178)]. Polymorphisms of the MR are also implicated but to a lesser extent [reviewed by Spijker and van Rossum (179)]. Evidence that early-stage AD patients have increased basal plasma cortisol levels (122, 123) suggests failure of the normal GR-mediated homeostatic feedback mechanism to maintain normative cortisol levels. Also, AD patients have decreased sensitivity to low-dose DEX suppression (124, 125). In mild to moderate AD, a progressive degeneration of HPA-axis is observed as serum cortisol levels remain significantly elevated together with DHEA and androstenedione levels, while estradiol levels were unaffected in females (126). One study reported that the GC-binding domain of GRα is not altered in AD, but other regions of the gene have not been investigated (180), nor has the GRβ isoform been examined. A study of post mortem brain samples by Gil-Bea et al. (55) state that the APOε4 genotype correlated with an abnormal increase in MR but not GR expression in BA10 of the frontal cortex (55). (Note that GR expression was significantly decreased regardless of APOe4 genotype for all AD brains.) The cause of an imbalance in GR/MR ratios is very likely to be the sheer presence of soluble Aβ protein. A single i.c.v. injection of Aβ(25–35) administered to rats is sufficient to cause an imbalance in GR/MR ratios in the hypothalamus, hippocampus, and amygdala (121). As a consequence, animals developed generalized dysregulation of the HPA-axis with increased CRF, ACTH, and cortisol levels. The behavioral profile of these animals also changed for the worse, with higher levels of anxiety as well as impaired short- and long-term memory (181). Extrapolating that data, in humans, it is likely that the early accumulation of the pathological soluble form of Aβ causes HPA-axis dysregulation by disrupting GR/MR-mediated homeostasis.

Young 3- to 4-month-old 3xTg-AD mice have increased hippocampal levels of MR and GR mRNA concurrent with Aβ accumulation (133). The increase in gene expression of both GR and MR is consistent with the post mortem findings of Gil-Bea et al. (55). There was also increased GR gene expression in the PVN of the hypothalamus; however, there was a marked absence of detectable Aβ pathology in the PVN that would be suggestive of a more generalized dysregulation of the HPA-axis originating at the hypothalamus. It would therefore be interesting to probe GR and MR expression levels in human post mortem samples. Consistent with increased GR expression was the concomitant decrease of CRH gene expression in PVN neurons. Previously, another study of the TgCRND8 APP transgenic mouse model had reported a similar progressive dysregulation of the HPA-axis, although the dysfunction was specifically attributed to adrenocortical hyperactivity that was detected in male prior to female Tg mice (182). As mentioned previously, subtle differences in the manifestation of depression and HPA-axis pathophysiology within the AD population have yet to be thoroughly investigated. That sex differences are also observed in preclinical models surely provides the impetus for further research, possibly starting with an examination of adrenocortical function. At the present time, a thorough examination for evidence of Aβ accumulation in adrenal cortical cells has yet to be conducted. It is possible that chronic adrenocortical hyperactivity is a feature of early AD, and the HPA-axis maintains homeostasis by downregulating GR and CRH. In a study by Murialdo (122), AD subjects showed higher cortisol and cortisol/DHEAS ratios and lower DHEAS levels in comparison with controls following the DST (183). Interestingly, 32% of AD subjects showed cortisol levels above the conventional cut-off of 140 nmol/L (DST non-supressors), despite no significant differences in the clinical parameters compared to DST suppressor patients. Both ACTH and cortisol levels were not different in suppressor and non-suppressor patients, but DHEAS levels were significantly lower in non-suppressor cases. This initial evidence is suggestive of adrenal specific pathology as another factor in AD-associated HPA-axis dysfunction. The precise temporal sequence of HPA-axis pathology is yet to be determined, but could potentially uncover novel biomarkers of early AD, or more specifically identify individuals with AD who are at risk of developing co-morbid depression.

### Glucocorticoid-mediated effects on AD molecular pathology

Thus far, we have discussed co-morbid depression as a behavioral manifestation of HPA-axis dysfunction (likely) caused by the accumulation of Aβ. Here, we will summarize the current evidence that the consequential hypercortisolemia in turn negatively modulates AD-related disease processes. Taken together, it emphasizes the importance of addressing HPA-axis dysfunction and the timely treatment of depression in AD.

Neuronal, astrocytic and mitochondrial metabolic disturbances (184–186) are consistently reported in various models of AD, and thus appear key to the disease process. Independently, those disturbances in cell energetics are similar to those reported in MDD (187–189), and each has been separately shown to be possible outcomes for chronic or excessive GC exposure (190–195). Thus, it is possible that many pathological features of AD are directly caused by the accumulated effects of increased GC levels. This is one aspect of AD pathophysiology that has yet to be thoroughly investigated. In this section, we will review the evidence that elevated cortisol levels as a result of HPA-axis dysfunction could significantly modify AD-related pathology and potentially hasten disease progression.

Perturbation of normal cell metabolism within the hippocampal network is another likely cause of the cognitive deficits and pro-depressive behaviors in AD. GC-induced metabolic changes are partially mediated by a key metabolic enzyme, adenosine monophosphate-activated protein kinase (AMPK). AMPK activity is crucial for normal functioning neuronal networks (196), and its enzymatic activity is prone to modulation by GC levels (197). A recent study used the AMPK inhibitor, Compound C, to correct the deficits in hippocampal LTP and LTD recorded from APP/PS1 transgenic mice (198). Examining the potential for Compound C treatment to correct HPA-axis dysfunction and rescue the pro-depressive phenotype of this particular AD transgenic mouse line would be an interesting follow-up. It should be stated that a disturbance of cellular metabolism is not a novel concept in the context of AD pathology. For example, abnormal homocysteine metabolism resulting in hyperhomocysteinemia is one aspect that has been heavily investigated. In their recent examination of post-mortem brains, Hooshmand et al. reported that elevated plasma homocysteine levels were significantly associated with the accumulation of neurofibrillary tangles and Aβ accumulation (199). That finding is supported by preclinical evidence associating hyperhomocysteinemia with the development of Aβ-related cerebral angiopathy (200). Independently, in rats, restraint stress induces elevated GC levels and also homocysteine levels (201). Therefore, it is also possible that abnormal homocysteine levels in AD are yet another consequence of HPA-axis dysregulation. However, the use of plasma homocysteine levels as a biomarker for co-morbid depression in AD remains to be validated (202, 203).

In a discussion of GC modification of AD pathology, it is impossible to ignore the contribution of stress. Given the evidence that HPA-axis dysregulation occurs in the earliest stages of AD, one could speculate that the further imposition of stress on an individual afflicted with AD could potentially hasten disease processes and also be a major factor in the development of depression [see review of Dong and Csernansky (204)]. A central theme of many studies in this area is understanding how the experience of stress impacts on *in vivo* Aβ processing. In rats, both exposure to chronic unpredictable stress and GC treatment resulted in the misprocessing and accumulation of Aβ levels, as well as hyperphosphorylation of tau protein, in the frontal cortex and hippocampus (205, 206). This impact of GCs on Aβ accumulation is also reproduced *in vitro* with rat neuronal PC12 cells (207). Those molecular pathologies were associated with the emergence of anxiety behavior and impairment of spatial memory in the Morris water maze. Mouse studies have also demonstrated that stress acting through the HPA-axis compounds, the accumulation of AD-relevant proteins. In the Tg1276 mouse model of amyloid precursor protein (APP) pathology, a single acute bout of restraint stress increased interstitial fluid Aβ load (208). The concentration of soluble extracellular Aβ is highly relevant to disease pathology. It is well accepted that the soluble form of the protein that exerts a neurotoxic effect, with one mode of mechanism possibly involving neuronal microRNAs (209). Curiously, there is a dearth of literature examining the progressive and cumulative effects of chronic mild stress. It is unrealistic to extrapolate data collected of a rodent that has been exposed to a single bout of stress to humans who experience a lifetime stress, each of varying degree. While it does not diminish the importance of establishing the pathological consequences of a single stressful event on Aβ levels in an *in vivo* model, the logical follow-up experiment would be to study the accumulated effects of chronic subthreshold stress, e.g., using the well-established paradigm of chronic mild stress. An experiment such as this would more closely model the persistent exposure to stress by humans and enable us to better understand how chronic stress impacts on AD disease processes.

In rats, manipulating the concentration of circulating corticosterone alters the severity of Aβ-induced neurodegeneration (210). The potential for stress to change soluble Aβ concentrations in humans has yet examined. This could be investigated through a longitudinal study to correlate the accumulation of stressful events with soluble Aβ load. Such a study could further examine the effect of manifesting depression, as well as the influence of risk genotypes. Interestingly, the stress-triggered increase in Aβ concentration can be inhibited by CRF receptor antagonists but not by corticosterone (208). That implies that hypothalamic and/or pituitary neuropeptides have the ability to directly regulate the production of Aβ protein. This finding opens up the possibility of novel targets within the HPA-axis for pharmacotherapy with the aim of minimizing Aβ build-up.

In addition to accelerating the accumulation of Aβ, other AD-related disease processes likely to be influenced by stress and abnormally elevated cortisol levels include the build-up of beta-amyloid plaques and insoluble tau inclusions (211–213). The impact of stress is not limited to neurons and also extends to astrocytes, which respond by upregulating APP and BACE1 (214). It is interesting and important to note that chronic corticosterone administration does not mimic the pathological effects of restraint stress (213). That suggests a dissociation and the existence of a more complex relationship between the broader cortical regions that regulate negative emotionality associated with the experience of stress, and the HPA-axis itself. Despite the challenges of discovering selective compounds and targeting precise brain regions, the HPA-axis remains an attractive physiological system to target for anti-depressive and possibly AD treatments. One particular target within the HPA-axis that has emerged is the CRF receptor. For example, in two different transgenic mouse models of AD, the pre-stress application of a CRF receptor Type 1 (CRFr1) antagonist prevented the stress-induced accumulation of Aβ, tau aggregation, neurodegeneration, and memory impairments (213). In a separate study, reducing CRFr1-singalling normalized the hypercortisolemia and anxiety phenotype of the APP/hAβ/PS1 knockin mouse model of familial AD (215). These are very promising outcomes and further investigations of CRFr1 antagonism would extend to examinations of HPA-axis function, as well as its anti-depressive potential.

It is reasonable to question why, despite the strong evidence of GR dysfunction and hypercortisolemia, direct intervention of GR function has not been adopted as widely available therapeutic option. It is not as straightforward as attempting to address the hyperactive response of the HPA-axis through administration of GR antagonists, e.g., DEX, RU486. Also, multiple side effects have been revealed from preclinical studies. One study found that while administering a 5 mg/kg dose of dexamethasone to Tg2576 transgenic mice decreased phosphorylation at specific residues of the tau protein, this also led to an increase in soluble Aβ(1–40) peptide concentration in the brain, which was associated with memory impairment (216). In contrast, 3xTg-AD mice treated with mifepristone (RU486) were found to improve on their cognitive deficits and have reduced Aβ levels and tau pathologies (217). The different outcomes of DEX and mifepristone treatments are not surprising despite being reliant on similar mechanisms of action, as evident from the studies of other conditions (218). Short-term treatment with mifepristone is effective in psychotic major depression (219) but its efficacy for treating co-morbid depression in AD is less certain (220). Therefore, further studies are required to probe the potential of early RU486 administration and its dosing regime as a treatment for depression in AD, as well as to elucidate other methods of modulating HPA-axis activity with minimal side effects.

## Depression in Parkinson’s Disease

Parkinson’s disease is a progressively degenerative neurological condition and the second most common neurodegenerative disorder after AD (221). It is most noted for the obvious motor abnormalities such as tremor, rigidity, and postural imbalance. However, non-motor symptoms including depression are commonly reported in PD patients (222, 223), prior to the onset of motor symptoms (22, 224), and with increasing frequency as the disease progresses (225). For example, disturbances in sleep and circadian patterns have been reported in newly diagnosed PD patients (226). Anhedonia, a lack of interest in novelty and pleasurable activities, has been hypothesized to be attributable to PD-related dysregulation of insular cortex activity (227). Schuurman et al. concluded in a retrospective study that having depression may induce the subsequent development of PD (228), although that could perhaps reflect the hastened trajectory of PD processes in the presence of depression.

The frequency of depression in PD has been cited anywhere between 2.7 and 70% (229) and this disparity is likely due to methodological differences. One of the earliest studies of this was by Mayeux et al. who reported 40% incidence of depression from 49 consecutive patients (230). Consistent with that figure, and at about the same time, Gotham et al. reviewed 14 studies and reported a mean frequency of depression of 46% (231). Several years later, Cummings found a similar level (~40%) in a review of 26 studies (232). More recently, in a Belgian cohort of 1086 PD patients, 15.6% presented major depressive episodes (233). In a smaller study of 202 PD patients, Hu et al. found 37.3% prevalence of depression (234). In contrast, Tandberg et al. concluded that of the 245 PD patients examined in their study, only 7.7% met the clinical criteria for MDD, although a further 45.5% were classified as mildly depressed (dysthymia) (235). Aarsland et al. utilized the Neuropsychiatric Inventory to screen for a variety of symptoms and found that 38% of 139 PD patients in Norway had depression (21). More recently, in a UK study, Schrag et al. reported that 19.6% of 92 PD patients had moderate to severe depression (236), so that estimate is likely to be even greater if those with mild depression symptoms were included. In a Brazilian study, Carod-Artal et al. found 47% of a cohort of 115 PD patients scored 11 or more on the Hospital Anxiety and Depression Scale, and concluded that this is a main determinant of quality of life for patients (237). Although the use of different instruments to diagnose depression between studies makes it difficult to compare figures, it is a reasonable conclusion that depression is more prevalent within the PD community than the general population.

Depression is often not identified and treated early in PD (234, 238). Mutations in the parkin (PARK2) gene are risk factors for early onset PD and confer higher risk for depression (239). There seems to be a missed opportunity to treat this symptom early in the disease, especially since there is no apparent PD-related inefficacy of treatment with common antidepressant drugs (240, 241). While there has been one study linking the presence of depression with abnormal gait (225), there is no strong evidence to suggest that depression significantly impacts the rate of progression of the motor symptoms or age of onset of the disease (unlike for AD as discussed in the previous section). However, PD patients with depression are reported to have greater severity of illness as rated by the Unified PD Rating Scale, and also present with other co-morbidities such as anxiety and memory problems (242). Increased apathy, itself one of the key diagnostic features of depression, has been linked specifically with a greater decline in cognitive performance of drug-naïve PD patients without depression (243). That finding suggests that apathy should be regarded as an independent neuropsychiatric feature of PD and be excluded as one of the diagnostic criteria for depression for PD patients. Furthermore, it could be more closely related to cognitive impairment and revealing of hippocampal dysfunction rather than the development of depression. To further support this dissociation of apathy from the symptoms of co-morbid depression, it had previously been suggested that treating depressive PD patients with SSRIs could in fact worsen their apathy scores (244). Further research is required to resolve the pathological basis of apathy and its relation to other symptoms of PD.

Since memory impairments and depression are closely associated with deficits in hippocampal function, this is one region of the brain that deserves closer scrutiny for the purposes of diagnosis or treatment. This is supported by a recent MR-imaging study that uncovered a significant negative correlation of severity of depression (Beck depression index scores) and bilateral hippocampus volume (245). In addition, a negative correlation also existed for amygdala volume. Together, it suggests that extrastriatal structural pathology accounts for the non-motor symptoms of PD. The findings of this study could be used as a basis for a future longitudinal study which tracks the progression of structural pathology in PD patients with and without depression. Armed with that information, it would then be possible to use the regression of hippocampal (and amygdala volume) as a predictor for the patient developing depression. Such an approach is not novel, as the rate of hippocampal volume change has previously been used as a predictor of mild cognitive impairment and dementia (246). Subsequently, profiling the changes to hippocampal volume could be used as a quantitative measure of treatment efficacy for interventions targeting cognitive and depressive symptoms in PD.

Interestingly, other personality traits closely associated with depression, such as neuroticism, have also been suggested as potential risk traits for developing depression in PD (247). Recently, depression was found to be the most important factor associated with suicidal ideation in PD (248–250). This is a serious psychological aspect of PD facing physicians and caregivers but one which could potentially be lessened through timely treatment of the depressive symptoms. Currently, the true extent to which manifesting depression alters the trajectory and severity of PD symptoms is not conclusive. However, given some evidence that specific symptoms might worsen in tandem with depression, it will be important to identify and understand the subtle differences in pathology related to the presence of depression in PD.

The use of animal models of PD to demonstrate a link between disease pathology and the manifestation of depression in PD has been somewhat limited. Most sufferers of PD have idiopathic PD, but ~15% of PD patients have a first degree relative with the disease, which suggests some degree of heritability (251). Several genes have been identified as familial risk genes for PD including α-synuclein gene (252, 253). There are conflicting reports of the behavioral phenotype of the A53T transgenic mouse model of PD (containing the human A53T α-synuclein associated with an autosomal dominant PD). One study found that these mice exhibited increased levels of anxiety and progressive cognitive deficits in addition to motor impairment (254). However, there have also been several studies reporting that overexpression of A53T α-synuclein results in reduced anxiety and hyperactivity (255–257). Currently, a thorough examination of a depression-related behavioral phenotype related to α-synuclein overexpression has yet to been reported. Interestingly, A53T transgenic mice have impaired hippocampal neurogenesis, which is corrected by chronic administration of the SSRI fluoxetine (258). However, that study did not examine treatment effects on a possible depression phenotype of that transgenic line. That remains a critical aspect of the A53T model to investigate because fluoxetine failed to correct the pro-depressive behaviors in the rat 6-OHDA model of PD (259). By comparison, transgenic mice lacking the CD157/BST1 gene (a risk locus in PD) do not develop motor deficits, but display behavioral responses indicative of increased depressive behavior (increased immobility times in the tail-suspension and forced-swim tests), increased anxiety and decreased preference for novel social interactions (260). The difference in behavioral phenotypes of these two distinct transgenic mouse models of PD suggests that specific genetic risk factors may be linked to a greater likelihood of developing depression in PD. This intriguing possibility has yet to be examined within a clinical population, and a preliminary retrospective examination of the currently available databases could uncover evidence of this.

Besides genetic risk factors, there is evidence that the primary pathological features of PD itself are sufficient to give rise to a depression phenotype. Rats treated with 6-OHDA to mimic the selective cell death of dopaminergic neurons in the striatum recorded greater immobility time in the forced-swim test for depressive-like behavior (261). However, that result could be simply explained by the impairment to the animals’ swimming ability. Consistent with that, one recent imaging study had reported that un-medicated PD patients with depression have lower 18F-fluorodopa uptake in the striatum compared to the patient group without depression, suggesting a greater degree of dopamine dysfunction within that brain structure (262). Resting state fMRI has also been used to show that PD patients with depression have increased neural activity in the orbitofrontal area but reduced connectivity of the prefrontal-limbic neuronal network (263). These initial findings are the basis for further studies, which aim to identify novel imaging-based biomarkers of depression in PD. Uncovering the genetic risk factors and mechanisms underlying the development of depression in PD will be important for the development of more effective treatments targeting the non-motor symptoms of PD. In the next section, we will examine the evidence and focus on pathology the HPA-axis as a common mechanism to co-morbid depression in PD.

### Dopaminergic dysregulation is central to HPA-axis pathophysiology in PD

The primary pathophysiology of PD relates to the abnormal accumulation of α-synuclein and formation of Lewy bodies in the brain (264), with good evidence that neuronal dysfunction occurs prior to cell death (265). However, whether Lewy bodies are causative or protective of cell death remains controversial [see reviews by Obeso et al. (266) and Schulz-Schaeffer (267)]. Despite the broad presence of Lewy bodies in cortical and limbic structures, there appears to be selective vulnerability and death of dopamine-secreting cells in the substantia nigra pars compacta (SNc) (268, 269). It is therefore reasonable to speculate that the presence of Lewy bodies could be a reliable predictor of the specific brains regions or physiological systems which are impacted upon in PD.

Interestingly, selective vulnerability is also observed in the hypothalamus wherein the neurosecretory cells of the PVN in the hypothalamus remain relatively free of Lewy body formation (270). At present, it is unclear why these neurons are capable of preventing the abnormal aggregation of α-synuclein. The extent to which the normal activity of CRF-immunopositive secretory neurons is impacted in PD is unknown due to a lack of studies. No recent studies have examined the status of CRF secretion in the PD brain and the literature is limited to one study from decades ago reporting of a reduction in CRF-like immunoreactivity post mortem neocortical PD brain tissue (271), and another which examined correlations of several neuropeptides including CRF in the CSF of idiopathic PD patients but without comparing actual concentrations to the control group (272). However, regardless of that future studies find, the cause of any hypothalamic pathology currently appears to be independent of α-synuclein. This is evident from a study of the 1-methyl-4-phenyl-1,2,3,6-tetrahydropyridine (MPTP) model of Parkinsonism which models selective dopaminergic cell death in the substantia nigra (273). In this study by Huang and Lee, targeted ablation of dopaminergic neurons in a separate brain region was sufficient to cause selective reductions of CRF-positive neurons in the PVN (and the central nucleus of the amygdala). That suggests that the survival of hypothalamic CRF neurons is regulated by dopaminergic pathways. Therefore, studies of dopamine-based interventions (e.g., l-Dopa replacement) could consider central measurements of CRF as an additional marker of treatment efficacy.

In contrast, the presence of Lewy bodies has been very recently confirmed in the posterior pituitary lobe (274), which is involved in the storage and secretion of antidiuretic hormone and oxytocin. The extent of pathology within the anterior pituitary is unclear which is unfortunate since the anterior lobe is where the ACTH-secretory neurons are located and is of specific relevance to this review. However, PD-related dopaminergic deficits are likely to disrupt normal pituitary function and ACTH secretion since the expression of proopiomelanocortin (POMC; the precursor of ACTH; the other being melanocycle-stimulating hormone, MSH) is directly regulated by the dopamine D2 receptor (275). In support of this, plasma ACTH levels of untreated idiopathic PD patients are significantly lower compared to healthy controls (276), consistent with the diminishment of dopaminergic signaling in the PD brain. Understanding how pituitary dysfunction could be an additional modulator of disease progression and symptom severity will warrant further research.

Compared to the available evidence of central HPA-axis pathology, evidence of peripheral pathology is scarce. Lewy bodies are also detected been detected in the adrenal gland, as well as the peripheral autonomic nervous system, including the heart and GI tract (277). Whether the accumulation of Lewy bodies directly impacts on adrenal function (for both adrenal cortex and medulla) is unknown at present. The collective evidence indicates that there is wide-spread dysregulation of the HPA-axis across multiple levels in PD. This is likely to underlie the high incidence of co-morbid depression in this disease. The progressive accumulation of HPA-axis pathology is unknown and will require further research. That knowledge could then inform us about how HPA-axis dysfunction influences the progression of PD symptoms, both motor and non-motor. For example, it has been proposed that targeting POMC expression could be a feasible therapeutic option in PD in future (278). A targeted intervention to correct or minimize HPA-axis dysfunction could prove to be highly effective in treating depression, in combination with more the more standard antidepressant medications.

While it is well regarded that ACTH and cortisol levels are altered in PD patients, there are conflicting reports of the directionality of the changes. Early studies had reported reduced levels of plasma ACTH and cortisol in untreated PD patients (276, 279). However, in a 24-h profiling study of plasma collected from 12 patients, Hartmann et al. found higher cortisol concentrations in PD patients, which they attributed to greater adrenal gland burst activity (280), concurrent with significantly diminished diurnal variation of cortisol levels. Similarly, elevated serum cortisol levels were reported in a larger collective study of idiopathic and non-idiopathic PD patients compared to controls (281). It is important to note that cortisol concentrations measured from saliva samples do not reflect any signs of pathology (282) and this should be a key consideration in the design of future clinical studies. While the inconsistencies between previous plasma profiling studies could be attributed to age, progression of disease or drug treatment, salivary cortisol levels appear to be unaffected by levodopa or dopamine agonist drug treatments (283), and do not correlate with duration of the disease and motor symptoms (282). Interestingly, one study reported that salivary cortisol levels could predict which PD patients were more likely to engage in risky behavior (284) and this could be an important consideration in the context of cognitive deficits or suicide ideation. The collective evidence supports the occurrence of a dysregulation of HPA-axis activity in PD based on abnormal cortisol levels, and the challenge at present is to identify the precise molecular pathology and develop effective treatment approaches.

The DST has previously been employed to demonstrate alterations in HPA-axis feedback signaling in PD. One early study stratified a small group of patients by the presence of depression and reported that PD patients with depression were more likely to be DST non-suppressors (285). Rabey et al. reported that a significant proportion of idiopathic PD patients were DST non-suppressors (17 of 32 patients; 53%) and had higher basal levels of cortisol and ACTH compared to healthy age-matched controls (20% non-suppressors) (286). That study also found that the proportion of non-suppressors was not altered after stratification of the patient group for dementia, suggesting that in PD, HPA-axis pathology is associated with co-morbid depression, not dementia. More recently, in a study of 11 PD patients, it was reported that all patients showed DEX suppression of serum cortisol levels (39). Interestingly, and as a demonstration of a potential link between cortisol levels and depression co-morbidity, those PD patients were provided with subthalamic nucleus deep brain stimulation over a period of 6 months, which led to improvement of depression symptoms correlating with reduced 24-h mean cortisol levels. Surprisingly, studies to-date have been limited to use the DST in isolation and have yet to attempt the DST in combination with CRH/ACTH application. However, these appear to be of low priority now because of growing evidence that HPA-axis dysregulation in PD is more reflective of disturbed central neurotransmission (discussed below).

There is increasing evidence to suggest that it is the broader PD pathology that impacts on HPA-axis activity. Volpi et al. compared the incremental response of ACTH and cortisol to administration of CRH and the non-specific serotonin receptor agonist fenfluramine in 10 PD patients who did not differ in basal ACTH/cortisol levels compared to controls (287). They reported that while CRH elicited similar ACTH/cortisol elevations in all subjects demonstrating intact functional response of the pituitary and adrenal glands, PD patients were non-responsive to fenfluramine administration. That indicated that serotonergic control of pituitary-adrenal response was disrupted; but it is still not clear whether the reported disturbance is due to impaired pituitary response to serotonergic signals or a loss of serotonergic regulation of hypothalamic CRH secretion. Substance P is well established to play a central role in the selective degeneration of dopaminergic neurons (288), and PD patients also exhibit an abnormal blunting of their ACTH/cortisol response to Substance P infusion (289). Administration of the opioid antagonist naloxone also elicits significantly blunted ACTH and cortisol responses in PD patients (290), but this impaired response has shown to be rescued after 1 year of levodopa/benserazide treatment (291). The successful treatment indicates that HPA-axis dysregulation in PD is partly due to disrupted opioid receptor signaling as a result of the loss of central dopaminergic neurotransmission in the PD-affected brain. While levodopa administration is known to acutely reduce cortisol release in patients (292, 293), the chronic effects of l-Dopa treatment on HPA-axis functionality have not been thoroughly investigated. It will be important to understand whether a partial benefit of long-term impact of chronic l-Dopa treatment is normalization of HPA-axis function and possibly preventing the manifestation of depression.

### Molecular pathologies potentially impacting on HPA-axis in PD

Compared to AD, there are comparatively fewer studies of the molecular pathology within the HPA-axis in PD. It is only very recently that imaging studies have reported volumetric changes and disrupted neurocircuitry of brain regions linked to emotionality and depression pathology, such as cerebral cortex and amygdala (245, 294–296). However, there is an absence of studies that critically examine HPA-axis function in PD. An initial study could examine the expression patterns of key signaling molecules within the HPA-axis in human post mortem tissue, e.g., visualization of CRF-immunopositive neurons in the PVN or even quantification of CRF mRNA levels in tissue samples. Such information is crucial in order to establish the progressive sequence of molecular pathologies of the HPA-axis in PD patients in relation to the development of depression. For this section, we will initiate discussion of the molecular pathologies by examining the hypothesized role of genetic polymorphisms with established associations with depression (e.g., dopamine D2 receptor, GR, and BDNF). We will also make mention of other factors that impact on HPA-axis function such as pro-inflammatory cytokines.

We will first consider the investigations of the dopamine D2 receptor polymorphism and its influence on PD pathology because dopaminergic dysregulation is at the core of PD pathology and the receptor plays a crucial role in the normal regulation of HPA-axis activity. Carriers of the A1 allele of the Taq1A *drd2* polymorphism have lower levels of D2 receptor binding (297) and this is implicated in depression symptomatology (298). Interestingly, this gene polymorphism does not appear to be a significant genetic modifier of depression on its own. Several studies have suggested that the Taq1A polymorphism acts through interaction with other genetic factors including the BDNF Val66Met polymorphism (299, 300). Within the PD population, it has been reported that the Taq1A *drd2* polymorphism does not influence treatment efficacy (301) but confers risk for PD that varies depending on ethnicity (302). Homozygous non-Hispanic whites having increased risk of PD compared to homozygous wild-type carriers. That is in contrast to African-Americans who are homozygous carriers having reduced risk of developing PD. Ethnicity-specific modification of PD risk is not unique and has also been reported for other genetic polymorphisms (discussed below). Presently, it is not known whether the Taq1A *drd2* polymorphism is sufficient to differentiate between depressed and non-depressed PD patients, and this should be a follow-up study for the future. Dopamine D2 receptor regulation of pituitary function and ACTH synthesis could potentially be impacted upon so physiological measurements of this should be taken.

Another monoamine-related polymorphism that is closely linked to major depression resides within the promoter region of the serotonin transporter gene (5HTTLPR), resulting in reduced expression of the serotonin transporter and dampened serotoninergic tone (303–305). This polymorphism is a risk factor for co-morbid depression in PD with patients bearing the short allele of the 5HTTLPR having significantly higher scores on the Hamilton Depression Scale (306, 307). In contrast, depression scores are not associated with the functional promoter polymorphism of the monoamine oxidase A (MAOA) gene, thus demonstrating functional relevance of the 5HTTLPR polymorphism to PD-associated depression. However, the conclusiveness of this association is not unchallenged (308). In support of the alternative, a radioligand binding study of 16 post mortem PD brains found no difference in serotonin transporter binding associated with the 5HTTLPR polymorphism (309). Validation of the association of this polymorphism with the increased risk of depression in PD will require further investigation utilizing larger sample sizes. Future studies could also examine whether the 5HTTLPR polymorphism is associated with greater severity of depressive symptoms within the PD population. In addition, taking cortisol and ACTH measurements should be strongly considered because separate research has found sex-specific modulation of basal cortisol and ACTH levels by the 5HTTLPR polymorphism (310, 311).

Surprisingly, there have been no studies of the GR polymorphisms in the context of PD symptoms or co-morbid depression. This is one area of research that is in dire need of development. The metabolism of other steroids could be involved in PD pathology, such as Vitamin D (specifically serum levels of 25-hydroxyvitamin D), which is significantly reduced in almost half of PD patients (312). Higher circulating 25-hydroxyvitamin D levels are associated with milder disease symptoms (312), while multiple polymorphisms in the 5’-end of the Vitamin D receptor gene are significant modifiers for age-at-onset of PD (313). There is no strong evidence to link Vitamin D insufficiency with HPA-axis dysregulation. However, Vitamin D remains a worthy candidate for further investigation as a therapeutic option for PD due to the strong evidence that Vitamin D metabolism exerts a significant influence over the progression of other neurological conditions such as multiple sclerosis (314) and supplementation with omega-3 fatty acids significantly reduces the risk of depression (315).

There is preclinical evidence to support the need for further research into the influence of functional polymorphisms of the GR. Rodent studies have shown that GC signaling is directly involved with the selective degeneration of dopaminergic neurons. Hence, this highlights the pressing need to fully understand the contribution of HPA-axis dysfunction and the subsequent elevation of cortisol levels to progression of PD pathology and symptoms. In a study to better understand neuroinflammation-related PD pathology, it was found that GR expression is downregulated in the substantia nigra post mortem, and that can be recapitulated in the MPTP mouse model of dopaminergic dysfunction (56). Closer examination of the MPTP model revealed that a loss of GR signaling was caused largely by an extensive nuclear re-localization of GR in activated microglia that caused an upregulation of pro-inflammatory genes. Taken together with clinical evidence of altered cortisol levels in PD patients, it is reasonable to speculate that dysregulated HPA-axis activity underlies the activation of microglia which is pro-inflammatory in the PD brain. Reactive microglia are found in the hippocampus of PD brains (316, 317). Interestingly, microglia that express MMP-2, an key inflammatory enzyme involved in breakdown of extracellular matrix, are exclusive to the substantia nigra and are not observed in the hippocampus or cortex (318), which suggests that there is specificity in the nature of neuroinflammation in the PD brain. It is also worth noting that TNFα levels are reportedly a distinct variable in accounting for severity of depression in a small cohort of 52 PD patients (as well as accounting for differences in their cognitive performance and sleep disturbances) (319). Recently, it was found that the TNFα initiates pro-apoptotic signals in dopamine neurons by inhibiting normal mitochondrial regulation of oxidative stress (320). This body of evidence supports on-going research into the use of anti-inflammatory and/or anti-oxidant compounds as additional therapies for PD (321–325).

While the Val66Met polymorphism of BDNF is strongly associated with major depression, several independent studies and meta-analyses have reported that this polymorphism does not increase the risk for developing PD and severity of symptoms (326–329). Interestingly, the findings of one meta-analysis study hint that ethnicity could be a significant modifier for the Val66Met influence with European, but not Asian, PD patients having a higher odds ratio of carrying at least one Val allele (330). The proposal that different genetic modifiers influence risk for PD and symptom severity is not novel, and the literature is awash with numerous studies conducted on specific ethnic populations. In general, the collective evidence supports the involvement of ethnic × genotype interactions in PD (331, 332). However, whether this extends to influence the development of depression by PD patients is unknown. Interestingly, recent studies have uncovered subtle cognition-related differences in PD patients associated with the BDNF Met allele (333, 334). Given that depression and cognition are intrinsically linked to similar brain structures such as the hippocampus, extrapolating those findings suggest the likelihood that risk or severity of depression could also be influenced by this common functional polymorphism of BDNF.

### Glucocorticoid-mediated effects on PD molecular pathology

As discussed above, disturbances of normal HPA-axis regulation are observed in PD. What is less clear is how a persistent dysregulation of GC levels impacts on PD disease processes. It is unclear if disease progression is accelerated in a patient manifesting co-morbid depression, resulting in poorer prognostic outcomes. Addressing those issues in a clinical population would be highly challenging, but possible to attempt with rodent models of PD. In this section, we will discuss the evidence implicating GCs in the worsening of PD-related pathology and discuss how these ultimately lead to an acceleration of symptomatology.

While age has long been considered the most prominent risk factor for PD (335), it is surprising that there has yet to be a comprehensive study of the added risk that stress (chronic or acute) confers. One study of British World War 2 prisoners of war (exclusively males) did not find any significant difference in cause of death attributable to PD (336). A more recent prospective study of almost 10,000 individuals found no significant relationship between psychosocial risk factors and the risk for PD (337). The authors collated self-reported information about major life events, economic hardship, social networks, and sleep/exhaustion levels. Interestingly, only sleep impairment was found to be significantly associated with PD-related hospitalization, lending further support to the crucial nature of hypothalamic dysfunction in PD symptomatology. Given the absence of a substantial body of clinical evidence, there is still great scope for future studies to more closely examine the potential role of stress in modifying the risk for developing PD.

Despite the lack of clinical evidence, the collective evidence from preclinical studies does strongly support the role of stress and altered GC signaling in influencing the development of disease pathology and symptoms. There is *in vitro* evidence implicating a role of disrupted GR signaling in PD molecular pathology. Administration of DEX to dopaminergic neurons disrupted GR signaling, resulting in an upregulation of α-synuclein protein levels (338). GC-mediated upregulation of α-synuclein levels has also been reported in peripheral blood mononuclear cells cultured from PD patients (339). Behavioral paradigms well established to induce physiological and behavioral stress in rodents such as food deprivation and tail-shock both worsen the severity of the motor deficits in the 6-OHDA lesion model of PD (340). Consistent with that, animals exposed to chronic restraint stress prior to the lesion develop more severe motor symptoms (341). As a demonstration that stress-induced changes in HPA activity and GC levels directly influence disease progression, corticosterone supplementation prior to the 6-OHDA lesion was sufficient to cause worsened motor symptoms. Furthermore, stressed animals do not display any level of spontaneous recovery from the lesion unlike unstressed animals. The GC influence is not specific to the nigrostriatal pathway and extends to brain regions such as the frontal cortex and hippocampus. Adrenalectomy-induced depletion of corticosterone in rats results in a dramatic downregulation of parkin protein in hippocampal neurons, and this effect is blocked by replacement of corticosterone (342). While the definitive experiment has yet to be conducted, this finding suggests that a stress-induced increase in GC levels would result in an upregulation of parkin expression and accelerate abnormal protein accumulation in PD. That hypothesis is supported by a recent report that mouse cortical neurons in primary culture upregulate parkin mRNA and protein levels following corticosterone treatment (343).

Negative environmental factors, such as stress, could also exert direct influence on PD pathogenesis by potentiating inflammatory responses. Stress-induced upregulation of pro-inflammatory cytokine levels is well established to be mediated by GC signaling. A single intranigral administration of LPS elicited a strong pro-inflammatory response and death of dopaminergic neurons, which was more severe in pre-stressed rats (344). As a clear demonstration of gene × stress interaction, transgenic homozygous knockout mice lacking the *parkin* gene do not develop nigrostriatal pathology until challenged with a low dose of LPS to initiate an inflammatory response (345). Hence, stress could be exerting a broad negative influence on all parkin-expressing cells in the brain. Future studies of how stress influences PD pathology should strongly consider utilizing this parkin knockout line in combination with stress-eliciting behavioral interventions. Extending similar research to compare distinct genetic models of PD could uncover novel gene × stress interactions that modulate the development of depression in PD, as well as the rate of disease progression. As mentioned previously, the A53T and CD157/BST1 transgenic mouse models of PD develop different behavioral features. It will be interesting to contrast the behavioral and physiological responses of both transgenic lines following exposure to stressors or corticosterone treatment.

Given that PD pathology involves multiple genetic disturbances, it can be expected that new insights into disease pathology emerge as evidence continues to accumulate. Lately, epigenetic modifications have emerged as significant molecular events in a variety of neurological conditions [see reviews by Urdinguio et al. (346) and Al-Mahdawi et al. (347)] and have been proposed to be useful for the development of novel therapies (348). DNA methylation is one class of epigenetic modifications, which is gaining in prominence within the PD research field (349). In a meta-analysis of PD genome-wide association studies with a pool of more than 10,000 PD cases, several new risk loci were identified (350). The methylation pattern at specific CpG dinucleotides in several genes expressed in the frontal cortex and cerebellum was significantly modified. Recent studies have reported that different brain regions exhibit different patterns of DNA methylation for specific genes (351). A specific DNA methylation signature of leukocytic α-synuclein (SNCA) and leucine-rich repeat kinase 2 (LRRK2) has even been proposed as a potential peripheral biomarker for PD diagnosis (352). Separate research supports stress and GC regulation of DNA methylation patterns (353). There have been numerous studies linking the exposure to stress with altered DNA methylation profiles of specific genes involved in HPA-axis regulation, e.g., CRF (354), major depression, and PTSD (355, 356). With the identification of a peripheral methylation-related biomarker of PD, it will be possible for future studies to explore the accumulation of stress and its influence on PD symptoms and co-morbid depression through longitudinal profiling of DNA methylation patterns.

## Depression in HD

Huntington’s disease is a monogenic, progressive neurodegenerative disorder that occurs in approximately 1 in 10,000 people in most Caucasian populations (357). A CAG trinucleotide repeat expansion mutation at the N-terminal end of the *huntingtin* gene on chromosome 4 leads to an expanded polyglutamine tract in the huntingtin protein (358, 359). In the post-mortem brains of HD patients, the presence of intranuclear inclusions containing N-terminal fragments of the mutant huntingtin protein as well as other proteins such as ubiquitin have been found (360). Significant atrophy is also present, most strikingly in the striatum and the cerebral cortex, although other areas, such as the hippocampus and hypothalamus, are also affected (361–364).

Depression is among the most common of the plethora of psychiatric symptoms present in HD, estimated to occur in between 20 and 50% of HD gene carriers (24, 25, 365). Almost 30% of HD patients attempt suicide at least once and death by suicide in the HD population is four to eight times that of the general population (366, 367). There is a high frequency of suicide ideation, and the presence of a depressed mood is a significant predictor to the level of suicide ideation (368, 369). Since the discovery of the mutation that causes HD and subsequent development of genetic screening technology, studies have shown that the psychological burden of discovering one’s HD gene status prior to motor symptoms manifestation can lead to great stress, which may contribute to the development of depression (370, 371). However, this mental stress alone has been found to be inadequate to explain the unusually high pervasion of depression in HD. For example, depression is estimated to be twice as common in HD as compared to Alzheimer’s patients (372) and PD (373). The suicide rate in PD is also much lower compared to HD (373, 374). More tellingly, HD gene carriers who were naïve to their gene status at the time of assessment and asymptomatic have higher prevalence of major depression than non-gene carriers who were equally at risk of inheriting the disease gene (375, 376). Importantly, correlation was also found between the severity and prevalence of depression and disease progression (377). A recent study that examined 1993 HD mutation carriers from 15 European countries using the Unified HD Rating Scale, found that 13% of subjects displayed moderate to severe level depression. The authors concede that this might be an under-report as healthy subjects were favorably chosen for the study. Interestingly, only 55% of those with moderate to severe depression were on antidepressants, suggesting under-diagnoses of this feature (378). The collective evidence indicates that depression is endogenous to HD pathophysiology and should be given special attention, particularly as it appears years before motor symptom onset.

In the general population, it has long been noted that females are twice as likely as males to develop clinical depression as well as generalized anxiety disorder, which is often co-morbid with depression (379, 380). Despite the extremely high prevalence of depression in the HD population, only very recently have studies examined whether sex differences exist. Investigating antidepressant usage in prodromal HD patients, Rowe et al. found that more female HD patients were prescribed antidepressants than males (381), an indicator of higher rates of depression. That finding was endorsed by another study reporting that female HD patients had significantly higher rates of past and current depression compared to male patients (382). These suggest that a similar sex difference is reflected in depression in HD. The only clinical study looking at sex hormones in female HD patients measured serum testosterone levels but conspicuously not E2 estradiol levels (383). Consequently, sex hormones may be involved in the mediation of depression in HD but this has yet to be confirmed.

Despite the high incidence of depression and similarities of its nature to clinical depression, very few studies have examined the effectiveness of antidepressant treatments in HD patients. The first such study occurred as recently as 2010, with a small cohort of 17 male HD patients who were given venlafaxine for 4 weeks, which resulted in alleviation of depression symptoms as measured by the Beck Depression Inventory and the Hamilton Rating Scale (384). It should be noted that no controls were used. Two controlled studies examined the effects of fluoxetine (385) and citalopram (386) but in non-depressed patients. Both studies found mild benefits to depression scores (Hamilton Depression Rating Scale). Both studies had low numbers of subjects (30 and 33 HD patients, respectively) and because non-depressed subjects were chosen, the potential benefits of the drugs tested remains unclear. There is therefore a pressing need for a large cohort, double-blinded, controlled study, ideally with multiple types of antidepressants and mood stabilizers to establish the best treatment for this serious symptom of HD.

### Hypothalamus pathology is the main mediator of depression in HD

Given the good evidence that co-morbid depression is frequent in HD, many studies have been conducted focusing on describing the symptoms of depression and treatment. Initially, it was thought that structural pathology of the hippocampal formation and shrinkage accounted for the cognitive deficits and depression in HD, with further speculation that this was caused by deficits in cell proliferation and neurogenesis [reviewed by Geuze et al. (387)]. However, post mortem investigations have uncovered no evidence of changes in the rate of cell proliferation in the subgranular zone of the hippocampus associated with HD (388). Also, there are no further differences between HD cases with and without mood symptoms. The human data are in contrast to the significantly reduced levels of hippocampal neurogenesis in R6/1 transgenic mice (389). However, subsequent studies have revealed that hippocampal synaptic plasticity is already impaired during the asymptomatic stages (390, 391). Thus, it is believed that neuronal dysfunction and disruption of the hippocampal neurocircuitry are the primary underlying factors for depression (and cognitive deficits) in HD. More recently, other disparate brain regions involved in emotional recognition such as the amygdala have been subject to study. HD patients have a specific impairment in recognizing facial signals of disgust, but the neurological basis for this is unclear. One study has suggested that the relative ability of HD patients to successfully recognize facial expressions of disgust and happiness correlated to anteroventral insular and amygdala volumes, respectively (392). Amygdala volume remains unaffected during the prodromal stages of the disease (393, 394), but undergoes progressive atrophy that is correlated to the severity of patients’ emotion-processing deficits (395). Serotonergic regulation of frontocortical activity plays an important role in emotional processing and is implicated in depression pathology (396). There is clinical (397) and preclinical evidence (398) that expression of a variety of serotonin receptors is reduced in HD, and these could contribute further to development of depression in HD. In contrast, less attention has been paid to identifying HPA-axis pathologies related to HD. There have been surprisingly few clinical studies, which have explored the more specific hypothesis that HPA-axis dysfunction could be the main cause of the greater incidence of depression in HD. In this section, we will summarize the evidence of HPA-axis pathophysiology in HD, focusing on the hypothalamus, and consider the clinical and preclinical evidence of altered neuropeptide signaling.

Similar to clinical depression, the first study to examine the HPA-axis in a small sample of 10 HD patients found evidence that the axis seems to suffer from abnormal regulation – higher basal plasma cortisol and ACTH were found in patients compared to controls (399). Leblhuber et al. examined 11 randomly selected male HD patients with definitive diagnosis and found higher serum levels of cortisol and reduced DHEAS compared to healthy controls (400). Most recently, pre-motor symptomatic HD patients significantly differed from advanced stage patients for measures of salivary cortisol, and this was associated with depressive symptoms (401). Interestingly, it was proposed that this association was lost in the advanced group possibly due to “exhaustion of the HPA-axis” and the prolonged exposure to elevated cortisol levels. In a study examining both the R6/2 mouse model and HD patients, the HPA-axis was hyperactive in R6/2 transgenic mice displaying advanced symptoms of the disease (402). Baseline corticosterone concentrations were elevated in this early onset mouse model starting at 5.5 weeks of age and this was attributed to hyperplasia of the adrenal gland, possibly arising from pituitary dysfunction since DRD2 expression was reduced. However, as eluded to above, young R6/2 mice displaying advanced symptoms of HD is not reflective of the wider clinical population. Thus elevated corticosterone level at this stage of disease progression in the model is not informative for potential correlations between HPA-axis state and early onset depression. The increased corticosterone level in the mouse model mirrored the elevated levels of cortisol found in urine samples of HD patients at later stages (stage III/IV) of disease progression. There was no significant difference between stage I/II patients and controls. Shirbin et al. have further proposed that non-depressed early stage HD patients may have a hypoactive HPA-axis but with the development of depression, hyperactivity of the HPA-axis occurs and masks pathophysiology by increasing cortisol levels similar to controls (403).

Abnormalities in the hypothalamus have long been implicated in HD given abnormal clinical alterations in energy metabolism, bodyweight, euglycemia and circadian rhythms (404–408). Importantly, Aziz et al. have found that night-time sleep impairment, likely the result of circadian alterations, correlated with depression (409). Examination of the diurnal regulation of cortisol secretion in early stage medication-free HD patients revealed evidence of HPA-axis-hyperactivity as overall cortisol secretion was increased, especially at specific periods such as the morning peak after awakening and during the early morning between midnight and 4 a.m. (40). These suggested a disturbance in the central GC feedback due to hypothalamic pathology. In a similar study, Van Duijn et al. measured salivary cortisol in pre-motor symptomatic, motor symptomatic, and control subjects across six time points, focusing on the morning peak in the first hour after awakening (410). They found that cortisol levels were elevated in presymptomatic HD gene carriers immediately after awakening but surprisingly no differences were observed between symptomatic patients and controls. However, the full period of the diurnal cycle was not examined and differences at other times, such as in the early morning, could have gone undetected.

The huntingtin protein is ubiquitously expressed by all cells so it is not surprising that in HD, there is reported pathophysiology at all levels of the HPA-axis – hypothalamus, pituitary and the adrenals. One approach to defining the hypothalamus as the key structure mediating the depressive symptoms in HD is to manipulate the extent of disease pathology and then observing its impact on the behavioral phenotype. Using a transgenic bacterial artificial chromosome (BAC)-HD mouse model which expresses the mutant full-length huntingtin gene and exhibits a pro-depressive behavioral phenotype, Hult et al. found that targeted inactivation of mhtt expression in hypothalamic neurons was sufficient to prevent the pro-depressive behaviors (411). The depressive phenotype of BACHD mice was successfully treated with sertraline treatment suggesting that HD-related depression is in part caused by disturbance of hypothalamic control of serotonergic tone. Also, BACHD mice do not develop deficits of hippocampal cell proliferation or neurogenesis (agreeing with the human PM data mentioned earlier). That is definitive evidence that hippocampal pathology alone does not cause depression in HD which is in contrast to its perceived role in MDD pathology. Using the R6/1 mouse model of HD which has a much milder disease progression compared to the R6/2, our group found that depression-like phenotypes (measured by the forced-swim and novelty-suppressed feeding tests) appeared in pre-motor symptomatic female R6/1 mice but not males or wild-types (398). The behavioral phenotype was initially correlated to changes in the expression levels of 5-HT receptors in the hippocampus and cortex. However, subsequently, we reported that hyperactivity of the HPA-axis was selectively observed in pre-motor symptomatic female mice but not males (412). This preclinical evidence implies that HPA-axis dysfunction is indeed more represented in female patients and thus, they may require special attention regarding diagnosis and treatment. Interestingly, although the female-specific dysregulation manifested as a sustained elevation of post-stress corticosterone levels, we found no differences in GR or CRF gene expression levels in the R6/1 hypothalamus. Instead, *in vivo* and *in vitro* evidence pointed to pathological regulation of corticosterone synthesis and secretion by the adrenal glands. Therefore, at least in the R6/1 transgenic model, adrenal pathology (despite the absence of adrenal hyperplasia) appears to precede hypothalamus pathology. Despite the mutant huntingtin protein and polyglutamine inclusions being readily detected in the adrenal gland (413), the physiological consequence of peripheral pathology which could possibly modulate the central neurological symptoms is often overlooked (414). Adrenal dysfunction can be gaged in HD patients using the DEX suppression test or the combinatorial DEX-CRH/ACTH stimulation tests, more typically used to diagnose Cushing’s syndrome or Addison’s disease. Pharmacological treatments targeting an overactive HPA-axis can then be applied such as mifepristone, a GR antagonist (415), which to the best of our knowledge has not been examined in either HD patients or rodent models.

### Molecular pathology of the hypothalamus in HD

Having discussed the physiological pathology of the hypothalamus as key to HPA-axis dysfunction and the depressive symptoms, this section will examine the molecular pathologies described of the hypothalamus. We noted that there is a somewhat limited body of human data expounding the molecular pathology potentially underlying the range of HD symptoms. However, studies of the various preclinical models of HD have been tremendously useful in uncovering novel molecular perturbations. These should be used to facilitate further clinical studies to confirm the pathologies predicted to occur in the human HD brain.

Structural and functional imaging studies have shown that hypothalamic aberrations are palpable even in early stage, prodromal HD gene carriers (362, 416, 417). Post mortem studies have found increased CRH mRNA expression in the PVN of HD patients (418), as well as reductions of orexin (419), oxytocin, and AVP expressing neurons (420). In contrast, a similar change in hypothalamic CRH gene expression is not evident in pre-motor symptomatic R6/1 HD mice (412). The possibility that this occurs as the disease progresses with age has yet to be explored. Many of the hypothalamic hormones and peptides have been shown to have modulatory roles on the HPA-axis such as oxytocin (421) and could potentially be applied clinically with little side effects as an adjunct or prophylaxis. Oxytocin has been shown to interact with estrogen receptor-β in the PVN to modulate both HPA-axis activity and depression-related behaviors of rats (422). Further studies could explore oxytocin as a candidate mediating the observed sex-specific nature of depressive behaviors in HD rodent models. This is especially pertinent as the hypothalamus also regulates the hypothalamic–pituitary–gonadal (HPG) axis, which has been shown to be dysregulated in HD patients (383, 423, 424) and rodent models (424–426). Recently, we reported that despite no changes to estrogen receptor expression in the hypothalamus, an acute injection of the estrogen receptor-β agonist diarylpropionitrile was sufficient to ameliorate the depression-like behavior of female R6/1 HD mice (427), suggesting that treatments based on modulation of estrogen signaling may prove to be fruitful.

Closely related to the altered diurnal rhythm of cortisol (see Dopaminergic Dysregulation is Central to HPA-Axis Pathophysiology in PD) are disturbances of diurnal patterns of activity and sleep–wake cycles that are reported by the early stages of HD (428, 429). These disease features are also recapitulated across several animal models of HD (430–434). Importantly, the disturbance of sleep patterns is a significant feature of major depression too (435–437), and involves altered gene expression of multiple neuropeptide receptors in the hypothalamus (438). The suprachiasmatic nucleus in the hypothalamus regulates circadian rhythms, which are significantly impacted upon in major depression [see reviews by Lall et al. (439) and McCarthy and Welsh (440)]. There is very limited evidence of hypothalamic molecular pathology based on the study of human HD brains. One post mortem cohort study found that immunoreactivity for vasoactive intestinal polypeptide (VIP) and arginine vasopressin (AVP) was significantly reduced in HD brains (441). The molecular pathology was selective as immunoreactivity for melotonin receptors was unchanged. A decrease in VIP expression is also observed in cortical layers of the human HD brain (442) and is reproduced in a mouse model of HD (443). Cell death is unlikely to be the primary cause of pathology in this brain region. The evidence suggests a disruption of neuronal signaling since the number of AVP-positive neurons remaining unchanged despite the reduced AVP mRNA levels and immunoreactivity (418). It is worth considering the prospect of using CSF vasopressin levels as a predictor of HD hypothalamic pathology since it has been used as an AD biomarker (444). In terms of treatment, agomelatine (a dual agonist of the melatonin receptors) is effective in treating MDD (445). This compound has yet to be trialed in HD patients, but recent preclinical evidence from a study of the 3-NP lesion model of HD is positive by rescuing weight loss, motor and cognitive deficits (446). Future research should comprise of testing this drug in genetic models of HD, as well as studying the effect of agomelatine treatment on related circadian disturbances.

The huntingtin protein is a key regulator of feeding behavior and body weight through its direct interaction with huntingtin-associated protein (HAP-1) (447, 448). A disturbance of feeding signals originating from the hypothalamus could account for weight loss in HD. Changes in appetite are frequently associated with depressed mood (449, 450) but a study specifically examining appetite levels has not been conducted of the HD population. It would be also interesting to gather clinical data of weight loss and attempt to differentiate between depressed and non-depressed HD patients based on changes in body weights. Disrupting the expression of HAP-1 alone is sufficient to cause HD-like hypothalamic degeneration in mice (451). On that basis, one could hypothesize that hypothalamic pathology in HD partly reflects a disruption of the normal interaction between the huntingtin and HAP-1 proteins. However, it was established early on that the HAP-1 protein interacts with the huntingtin protein in a manner that is independent of the abnormal polyglutamine tract and does not contribute to the molecular pathology as observed in transgenic HD mice (452).

In post mortem HD brains, mRNA levels of prohormone convertases are decreased in the hypothalamus (453). Prohormone convertases are essential for the regulation of neuropeptides such as NPY (454) but their expression and activity have not been a subject of active investigation in preclinical models of HD. Studies have shown that NPY-positive interneurons are a distinct population of cells, which are specifically impacted upon by disease processes (455). Therefore, despite the lack of knowledge of how NPY modulates the disease processes, it is not surprising that there is evidence of NPY exerting beneficial effects in preclinical rodent models by slowing the progression of disease symptoms including minimizing weight loss (456, 457).

In considering possible reasons for the somewhat limited body of information regarding molecular pathology of the hypothalamus, we suggest that it could either be due to greater focus on collecting physiological measurements of hypothalamic/HPA-axis dysfunction, or there is an investigative imbalance which sees the research focused on other brain regions (e.g., striatum, cortex, hippocampus). We are not understating the importance of establishing the physiological pathology of the hypothalamus since it is more applicable in the clinical setting, being used as disease biomarkers or measurements of treatment efficacy. Admittedly, markers of neuronal molecular pathology are not practical in the clinical setting with the exception of perhaps brain biopsies. However, such information is extremely useful, even vital, when using preclinical models to understand the precise timeline of disease progression. It would then be possible to reconstruct a precise disease timeline for distinct brain regions and signaling pathways. Armed with that, it is not unimaginable that future treatment strategies target multiple pathologies in multiple brain regions to address the wide spectrum of HD symptoms.

### Glucocorticoid influence on HD symptoms and pathology

Given the evidence of a hyperactive HPA-axis and upregulation of hypothalamic CRH gene expression, combined with the higher risk of depression, it is important to determine and understand how increased GC levels directly impact on HD pathology and symptom progression. Unfortunately, our present understanding of GC regulation of mutant htt protein processing or accumulation of protein aggregates is poor due to the absence of comprehensive *in vitro* studies of how GCs regulate *htt* gene transcription, protein translation, or the rate of nuclear inclusion accumulation. Thus, due to the lack of any clear evidence that GC signaling modifies the molecular basis for this disease, we will instead discuss the available clinical and preclinical evidence that stress constitutes a significant non-genetic modifier of HD.

However, we will start by highlighting two studies that have shed some light into the direct regulation of mhtt-mediated molecular pathology by GC signaling. A study conducted more than a decade ago reported reduced survival of non-neuronal cell cultures following the disruption of GC signaling due to decreased polyglutamine protein solubility and increased rate of nuclear aggregate formation (458). Follow-up studies are required to demonstrate that neuronal and non-neuronal cells process mutant htt protein differently to the normal protein, and this is directly influenced by GC levels. There have also not been any preclinical studies that have characterized the impact of stress on these measures of cellular pathology. However, by applying the GR agonist DEX to fly and mouse models of HD and reporting that this reduced protein aggregate load, one recent study has demonstrated the *in vivo* impact of GC regulation on disease pathology (459). There is still tremendous opportunity for future studies to more closely examine how htt (mRNA and protein) is regulated by GC signaling, how the kinetics of this is changed in HD, and how elevated GC signaling due to stress or HPA-axis dysfunction might contribute to depression and other disease symptoms.

By comparison, there have been numerous clinical studies investigating the genetic (460–463) and environmental (e.g., diet) modifiers (464) of the age-of-onset of HD. Strangely, there has yet to be a specific study of the potential impact of life-long stress or major traumatic life events on age-of-onset. Despite the convincing evidence of the higher risk for depression and HPA-axis dysfunction, it is odd that life-long stress or major traumatic experiences have not been considered to be disease modifiers since these are both significant factors for MDD. Focusing on preclinical models of HD, studies investigating stress as a potential modifier of disease symptoms are few in comparison to studies of diet (465–468) or environmental modifiers (469–473). The first study, which specifically set out to examine the impact of stress on HD symptoms, imposed daily restraint stress on asymptomatic young adult (8 weeks old) R6/1 transgenic mice until motor symptoms developed (474). Chronic restraint stress did not have a negative impact on motor symptoms, anhedonic behavior (saccharine preference), or short-term working memory (Y-maze). It was discussed that the lack of an observable effect on behavioral measures was due to the extended period of restraint stress and mice could be acclimatizing to the stressful experience. In order to address this possibility, Mo et al. directly manipulated GC levels by administering corticosterone through drinking water in order to induce sustained high levels of corticosterone in HD mice (475). They found that this experimental approach was sufficient to induce anhedonic behavior and diminished olfactory sensitivity in female mice after just 2 weeks, but the HD mice were not more sensitive to GC treatment. Interestingly, emergence of corticosterone-induced cognitive deficits was accelerated in male, but not female, HD mice compared to wild-type controls (474). While it is not surprising that corticosterone influences cognitive performance given that the high levels of GR expression in the hippocampus, the sex-specific manner in which this was observed is highly novel. This needs to be further investigated in a clinical population to determine if male HD patients develop cognitive deficits at an earlier age and if this translates to more severe cognitive deficits over the course of the disease. By comparison, corticosterone administration did not alter the age of onset of motor symptoms in HD mice, replicating the absence of impact by restraint stress and further indicating a specific influence of GCs on cognitive aspects of HD. Future work should investigate depression-related behavioral measures in preclinical HD models. The findings of these initial studies should also guide the design of future clinical studies investigating stress as a non-genetic modifier of disease symptoms by focusing on cognitive measures (and possibly depression) instead of motor symptoms.

One way by which increased GC signaling could modulate the cognitive deficits described in the R6/1transgenic model is through its regulation of BDNF/TrkB signaling. BDNF/TrkB signaling is essential for hippocampal synaptic plasticity and has a well-established role in cognition (476, 477). BDNF/TrkB levels are also posited to be involved with depression pathology and are diminished in post mortem studies (albeit based on suicide cases and having not been replicated by independent research groups) (478–481). mRNA and protein levels of both BDNF and TrkB receptor are reduced in post-mortem HD brain tissue (482–484). The link between BDNF/TrkB signaling, GCs, and depression has been thoroughly explored in rodent models and given much attention since stressors such as forced immobilization and social defeat consistently decrease BDNF/TrkB levels in the hippocampus, cortical and subcortical brain regions of rodents (485, 486). Corticosterone treatment recapitulates the stress-induced reductions of BDNF and TrkB expression in the hippocampus and frontal cortex in rodents (487–491). As a demonstration of the important role of TrkB in regulating GC influence on cognitive processes, stress-induced spatial memory impairments can be blocked by 7,8-dihydroxyflavone, a selective TrkB agonist (492). Interestingly, Mo et al. reported that the modulation of R6/1 cognitive deficits by corticosterone was associated with a specific increase in the levels of phosphorylated TrkB protein in the hippocampus with no changes to BDNF or GR expression (474). The selective TrkB agonist 7,8-dihydroxyflavone has been found to benefit motor symptoms and survival of the N171-82Q mouse model of HD (493) but studies of this compound as a therapeutic for cognitive deficits, depressive behavior or HPA-axis pathology have yet to be conducted. Besides TrkB, BDNF also signals down-stream through its low affinity p75NTR receptor which is increased in the caudate of HD brains (484). That observation is not too dissimilar from studies of the hippocampal tissue from suicided post mortem brains (480, 494). Therefore, it is also possible that elevated GC signaling (stress) impacts on HD cognitive and affective symptoms due to an imbalance of the TrkB/p75NTR ratio. Future post mortem studies to quantify phospho-TrkB and p75NTR levels should be conducted to determine if their relative ratios may be predictive of the severity of cognitive deficits, patients’ life-long burden of stress, depressed/non-depressed status or HPA-axis dysfunction.

Closely related to BDNF/TrkB signaling, antidepressant compounds such as the SSRI sertraline have been successfully used to alleviate the pro-depressive behavioral phenotype of several transgenic mouse models of HD in a BDNF-dependent manner (398, 472, 495–497). To-date, no study has examined whether the behavioral effects involve modulating hypothalamic and/or HPA-axis function by SSRIs. However, despite an initial promise of SSRIs possibly benefiting motor and cognitive symptoms based on preclinical evidence (389), the clinical evidence thus far indicates no significant benefit on any symptom apart from mood (385, 386). Interestingly, physical exercise, a potent stimulant of BDNF gene expression in the hippocampus (498), shows benefits on HD symptoms in preclinical (471, 472, 499) and clinical studies (500, 501). It is possible to quantify BDNF levels from patient blood samples, so future studies could consider attempting to correlate changes in peripheral levels of BDNF with exercise therapy to improvements in cognitive deficits and mood. Other parameters worthy of consideration include potential normalization of diurnal cortisol patterns, improvement of sleep, and normalization of DEX response. Preclinical studies conducted in parallel could also investigate whether exercise interventions have the capacity to correct HD-related HPA-axis dysfunction.

## Discussion

**Figure 1:**
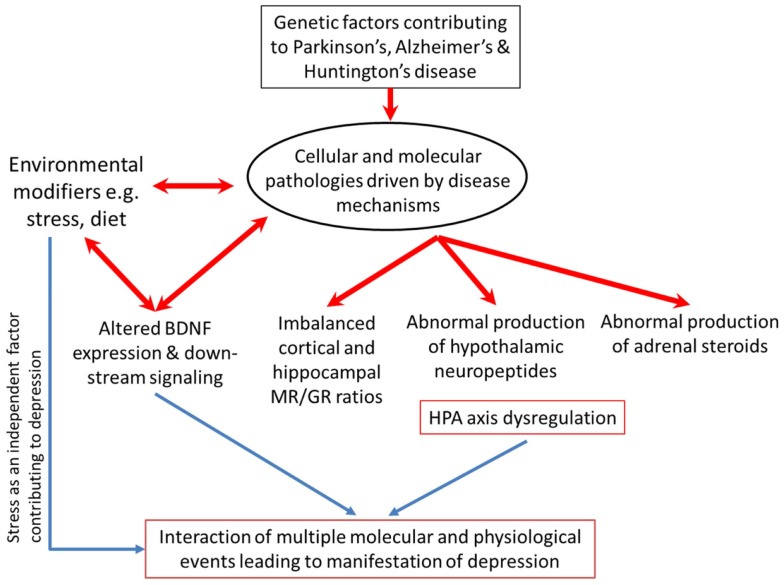
**Broad schematic of gene × environmental interactions potentially causative of the greater incidence of co-morbid depression in AD, PD, and HD**.

Alzheimer’s diseases, PD, and HD are neurodegenerative disorders mostly associated with their distinctive motor symptoms (PD and HD) or cognitive deficits (AD and HD). However, there is increasing evidence that the lesser-studied peripheral aspects of these conditions do exert significant influence on the severity of symptoms, the rate of disease progression, and thus the patient’s overall quality of life. In this review, we have focused on depression as a common co-morbidity of AD, PD, and HD. We discussed the higher prevalence of depression in all three diseases as a function of HPA-axis pathology by drawing upon clinical and preclinical data (see Figure 1 for broad schematic). In this review, we were struck by the lack of clinical evidence from large patient cohorts with respect to HPA-axis pathology. Detailed understanding of how stress and GCs impact on the molecular and cellular aspects of pathology is lacking for some. While data from cohort studies supported the greater incidence of depression, there was little by way of definitive functional assessments of the HPA-axis (presented as Tables 1–3). It is essential that such studies be performed in future so that the robustness of findings from smaller clinical studies can be independently assessed. Gathering data from larger cohorts would also enable investigators to examine for potential subtle differences in HPA-axis pathology specific to the disease in question. This is especially interesting given the complex nature of certain diseases, such as AD, which are linked with multiple genetic risk factors as each may contribute a different genetic load in dictating the extent of HPA-axis pathophysiology. While large cohort studies are expensive and require intensive investments of time and resources, they are necessary if the field is to bridge the current gap between the clinical and preclinical studies. Preclinical data inform us that specific pathologies may exist at distinct segments of the HPA-axis but ultimately, confirmation can only be gleaned from clinical evidence. The ability to match up preclinical and clinical data is powerful as it provides the impetus to further study the molecular aspects of disease using preclinical models. Furthermore, researchers will be able to use those preclinical models to assess therapeutic interventions with greater confidence for reproducing the beneficial effects in clinical environments.

Hypothalamic–pituitary–adrenal-axis dysregulation, just as depression diagnosis, can often be identified in the early stages of the diseases we elected to discuss. We propose that in addition to advocating for mandated monitoring for signs of clinical depression, regular clinical assessments of HPA-axis regulation should be considered. With growing awareness of depression as a diagnostic symptom (as in the case of HD), it is perhaps time to explore the potential of indicators of HPA-axis activity as independent disease biomarkers. Given the relative effectiveness of various pharmacological compounds and physical therapies to treat both depression and HPA-axis pathology, there is an opportunity to intervene at the earliest stages of disease. To effectively slow the rate of disease progression and to maintain a certain quality of life for the patient is highly attractive and definitely one worth considering.

## Conflict of Interest Statement

The authors declare that the research was conducted in the absence of any commercial or financial relationships that could be construed as a potential conflict of interest.
